# Chaotic fluctuations mark the sign of mental activity in task-based heart rate variability

**DOI:** 10.1038/s41598-026-43385-z

**Published:** 2026-03-24

**Authors:** Tomoyuki Mao, Hidetoshi Okutomi, Ken Umeno

**Affiliations:** 1 Department of Applied Mathematics and Physics, Graduate School of Informatics, Kyoto University, Sakyo-ku, Kyoto, 606-8501 Japan; 2 Technology Management Division, Toshiba Information Systems (Japan) Corporation, Kawasaki-ku, Kawasaki, Kanagawa 210-8540 Japan

**Keywords:** Nonlinear dynamics, Time series, Nonlinear phenomena, Information theory and computation

## Abstract

Heart rate variability (HRV), regulated by the autonomic nervous system, is typically assessed using standard time-domain and frequency-domain methods to evaluate autonomic function. However, conventional linear analyses capture only a limited aspect of HRV, as the human body, including the cardiovascular system, is intrinsically nonlinear. In light of this, there has been growing interest in nonlinear analyses grounded in chaos theory and complexity science. In this study, we conducted a comprehensive comparison of time-domain, frequency-domain, and chaos/complexity indices derived from R-R interval (RRI) analysis during both physical and mental tasks. The results clearly demonstrate a significant increase in chaos/complexity indices during mental tasks, while conventional indices remain unchanged—underscoring the unique sensitivity of nonlinear measures to cognitive processes. These findings highlight the relevance of chaotic dynamics and complexity in HRV as a valuable perspective for understanding brain-heart interactions. Furthermore, based on the experimental findings, we propose a new hypothesis, consistent with previous research, regarding the emergence of chaotic features in HRV during cognitive activity.

## Introduction

The autonomic nervous system is essentially involved in the regulation of heart rate. The sympathetic nervous system increases heart rate, whereas the parasympathetic nervous system decreases it. The fluctuations in the intervals between heartbeats resulting from these opposing actions are referred to as heart rate variability (HRV). A standardised method for HRV analysis was established in 1996^1^, enabling quantitative assessment of autonomic nervous system activity. However, both time-domain (statistical) and frequency-domain (spectral) analyses, which form the basis of conventional HRV analysis, are linear methods and therefore capture only certain aspects of HRV. The limitations of linear approaches have been widely discussed. Such approaches assume that heart rate time series are stationary, yet this assumption is often violated in real-world data^2,3^. Moreover, HRV signals are inherently nonlinear and complex, exhibiting dynamical features that cannot be adequately captured by conventional linear analyses^4^. Consequently, traditional linear techniques are frequently insufficient for describing the complex behaviour of HRV^2,3^. By contrast, nonlinear analyses have demonstrated superior sensitivity to linear approaches in several contexts^5–7^. Thus, conventional analytical indices provide a limited characterisation of HRV, and alternative, nonlinear methods may offer additional, valuable insights into the mechanisms underlying heart rate regulation.

Nonlinear analysis has recently attracted considerable attention as it offers a novel perspective distinct from conventional linear approaches^8^. The human body, and in particular the circulatory system, is widely regarded as a nonlinear system in which interacting regulatory mechanisms generate complex dynamics. For this reason, the application of nonlinear metrics, such as measures of chaos and complexity, to the analysis of HRV is a rational and promising approach. Importantly, nonlinear features of HRV are not static but vary with the physiological state, being attenuated under sympathetic dominance (e.g. during postural stress) and enhanced under vagal control^9^. This condition-dependence suggests that nonlinear HRV indices may provide useful biomarkers of cardiovascular regulation, adaptability, and health status. KUBIOS^10^, a widely used software package for HRV analysis, also incorporates several nonlinear indices alongside traditional linear methods.

Chaos was the first nonlinear characteristic to receive significant attention in this context. Chaos arises in nonlinear systems when the dynamics become so complex and unpredictable that they appear random, despite being governed by deterministic equations^11–13^. It has been proposed that such chaotic behaviour confers healthy physiological systems with the flexibility required to adapt effectively to a wide range of stimuli^14^. Evidence suggests that chaotic dynamics may be present in HRV^15,16^, and that the mechanisms underlying this behaviour may resemble those of neural network models, thereby contributing to heart rate adaptability and regulatory flexibility^17^.

Over time, additional perspectives—such as complexity and fractal properties—have emerged, leading to a wide range of research findings. For instance, during mental arithmetic tasks, increases in the conditional entropy of blood pressure and respiration have been observed, while the conditional entropy of the R-R interval (RRI) remained unchanged^18^. Conversely, RRI entropy has been shown to increase during cognitive tasks performed in the absence of stress-inducing external factors, such as environmental noise^19^. Furthermore, individuals exhibiting anxiety responses have been found to demonstrate significantly reduced HRV complexity under stress or anxiety, relative to those without such responses^20^. In addition, heart rate complexity has been suggested to correlate more closely with cognitive function and mood than traditional time-domain or frequency-domain HRV indicators^21^. Notably, nonlinear HRV analysis has revealed sympathetic hyperactivity in post-treatment cancer patients that was undetectable using linear methods^6^. Similarly, nonlinear approaches have shown greater effectiveness than linear methods in the early detection of cardiovascular abnormalities in patients with depression^7^ and in identifying psychological stress^5^. Collectively, these findings suggest that nonlinear HRV analysis offers enhanced sensitivity and diagnostic utility compared with traditional linear approaches.

Previous studies have reported somewhat divergent findings, largely attributable to differences in research objectives, study populations, experimental conditions, and analytical methodologies. Some investigations have examined healthy individuals, while others have focused on patients with specific diseases; moreover, the experimental settings have varied widely, encompassing diverse physiological phenomena. Such heterogeneity hampers direct comparison, synthesis of results, and the accumulation of consistent knowledge. In addition, although a range of indices is employed in nonlinear analysis, approaches grounded in chaos theory have become less common and are often overlooked. Yet chaos is a property observed universally across complex systems and may be fundamental to understanding the mechanisms underlying heart rate regulation. One contributing factor to the decline in chaos-based analysis is the absence of accessible indices that can be easily derived from heart rate data and interpreted mathematically with clear meaning. Addressing these limitations is critical for developing a more coherent and fundamental framework for nonlinear HRV research. At present, however, there remains no consensus on how the chaotic and complex properties of HRV should be interpreted, or which physiological states they represent. Consequently, nonlinear analytical approaches to HRV lack standardisation, and although they hold clinical promise, they have not yet been incorporated into routine practice.

In this study, we implemented a simplified experimental protocol involving both physical and mental tasks in healthy participants, with the aim of establishing a basis for comparing a range of linear and nonlinear HRV indices. We conducted a comprehensive comparison of conventional and nonlinear measures to examine the physiological states reflected by chaos/complexity indices. In addition, we introduced chaos degree (CD)^22,23^ and improved chaos degree (ICD)^24,25^, which are the measures proposed as information-theoretic indicators of chaos. Quantifying chaos from observed time-series data, such as RRI, is inherently challenging, particularly when the governing equations of the system are unknown. This challenge remains a key issue in chaos research, limiting the direct application of chaos analysis to real-world data. A notable advantage of CD and ICD is that they can be calculated directly from time-series data without requiring prior knowledge of the underlying system dynamics. As research in this area progresses, CD and ICD are emerging as practical quantitative indices of chaos. The present study therefore aims to bridge the gap between chaos research and HRV research. The findings of this study indicate that chaos/complexity indices of HRV significantly increase in response to mental tasks, whereas conventional indices exhibit little or no significant change. These results suggest that chaos/complexity indices may capture aspects of brain activity that are not reflected in traditional HRV measures.

## Results

**Fig. 1:**
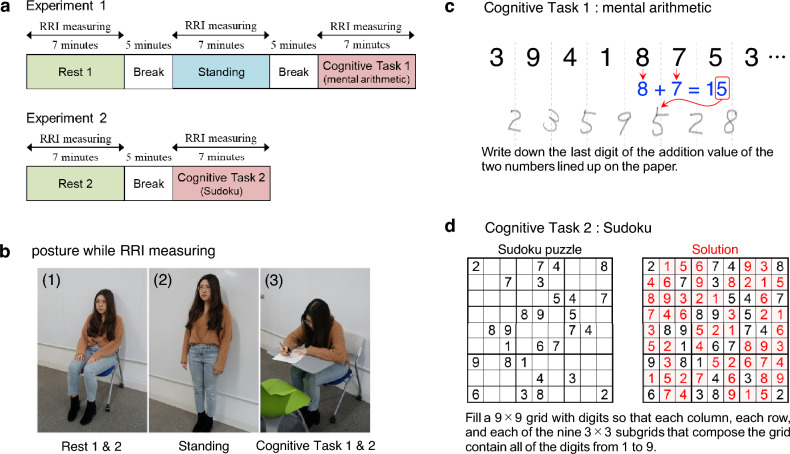
Figures to explain the experiment.**(a)** Procedure of measuring RRIs in Experiment 1 and Experiment 2. Participants took a 5-minute break between 7-minute RRI measurements in each condition.**(b-1)** Posture for measuring RRIs in the Rest condition.**(b-2)** Posture for measuring RRIs in the Standing condition.**(b-3)** Posture for measuring RRIs in the Cognitive Task condition.**(c)** Illustration showing the method of mental arithmetic performed in Cognitive Task 1.**(d)** Explanation and example of Sudoku performed in Cognitive Task 2.

**Fig. 2:**
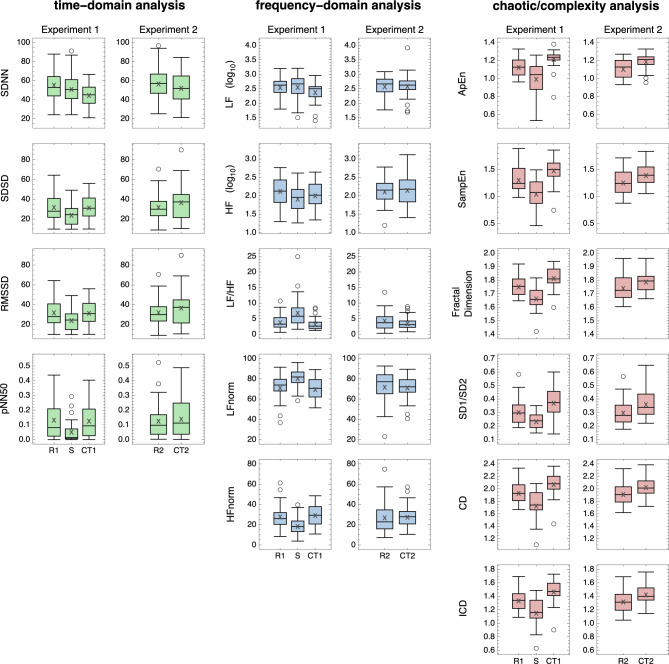
Box-and-whisker plot of the results of Experiment 1 and Experiment 2. Left side: The indices included in the time-domain analysis. Centre: The indices included in the frequency-domain analysis. Right side: The indices included in the chaos/complexity analysis. The number of data points (sample size) was 27. The horizontal axis labels are explained as follows: R1 represents Rest 1, R2 represents Rest 2, CT1 represents Cognitive Task 1, CT2 represents Cognitive Task 2, and S represents Standing.

We conducted two experiments to compare how the conventional time-domain and frequency-domain indices and the chaos/complexity indices of HRV are characterized in the mental and physical task conditions. The experimental procedure is outlined in Fig. 1a. In Experiment 1, the RRI was measured under three conditions: Rest 1, where no task was imposed; Standing, in which participants were required to maintain an upright posture as a physical task; and Cognitive Task 1, where mental arithmetic (Fig. 1c) was used as a mental task. The RRI data were recorded for 7 minutes in each condition, with a 5-minute break between measurements. Experiment 2 aimed to investigate whether similar characteristics were observed when the mental task was altered. In this experiment, Rest 2 was identical to Rest 1, with no task imposed, and Cognitive Task 2 involved the imposition of Sudoku (Fig. 1d) as the mental task. A total of 27 healthy participants took part in both experiments, completing 5 sets of each task. The recorded RRI data were comprehensively analysed using time-domain analysis, frequency-domain analysis, and chaos/complexity analysis, and the results were compared across 15 HRV indices listed in Table 1. For each participant, the index values calculated from the five repetitions were averaged prior to analysis. A box-and-whisker plot of all data from this experiment is shown in Fig. 2, with the means and standard deviations provided in Table S1. The within-subject means and standard deviations for each indicator, calculated across the five repeated measurements, are presented in Tables S2–S16.

**Table 1:** The indices of RRI analysis in Experiments 1 and 2. Upper 4 indices: The indices included in the time-domain analysis. Middle 5 indices: The indices included in the frequency-domain analysis. Lower 6 indices: The indices included in the chaos/complexity analysis.

Index	Units	Description
**Time-domain analysis**		
SDNN	ms	Standard deviation of all NN intervals
SDSD	ms	Standard deviation of differences between adjacent NN intervals
RMSSD	ms	The square root of the mean of the sum of the squares of differences between adjacent NN intervals
pNN50	–	The number of pairs of adjacent NN intervals greater than 50 ms / the total number of NN intervals
**Frequency-domain analysis**		
LF	ms$^2$	Power in low frequency range (0.04–0.15 Hz)
HF	ms$^2$	Power in high frequency range (0.15–0.4 Hz)
LF/HF	–	Ratio LF / HF
LFnorm	%	Ratio LF / (LF+HF) * 100
HFnorm	%	Ratio HF / (LF+HF) * 100
**Chaos/complexity analysis**		
ApEn	–	Irregularity rate in time series data
SampEn	–	Irregularity rate in time series data (improved method of ApEn)
Fractal dimension	–	Self-similarity in the time series data (Higuchi dimension)
SD1/SD2	–	Standard deviation along the minor axis in the Poincaré plot (SD1) / standard deviation along the major axis in the Poincaré plot (SD2)
CD	–	Degree of chaos calculated directly from time series data
ICD	–	Approximate value of the Lyapunov exponent calculated via CD

### Statistical significance test

Notable differences were observed in the results of statistical significance tests between (1) Rest 1 and Standing, (2) Rest 1 and Cognitive Task 1, and (3) Rest 2 and Cognitive Task 2, as summarised in Table 2. Overall, the following trends were observed. In the conventional analysis (time-domain and frequency-domain analyses), there were significant increases in indices commonly associated with sympathetic modulation and decreases in parasympathetic-related indices from Rest to Standing. In particular, LF/HF, often interpreted as reflecting sympathovagal balance, increased, while parasympathetic indices such as SDSD, RMSSD, pNN50, HF, and HFnorm decreased. In contrast, from Rest to Cognitive Task, only some indices showed a decrease, with no significant changes observed in the others. In the chaos/complexity analysis, however, the changes were more pronounced. Specifically, all six indices decreased significantly from Rest to Standing and increased significantly from Rest to Cognitive Task.

**Table 2:** P-value for each pairwise comparison calculated using the Wilcoxon signed-rank test; significant, with comparisons deemed significant at a $1\%$ threshold after Holm–Bonferroni correction indicated by an asterisk; and direction of change of the right side relative to the left-side reference, with upward and downward arrows indicating an increase or decrease in the mean index value, respectively. (1) Comparison between Rest 1 and Standing. (2) Comparison between Rest 1 and Cognitive Task 1 (mental arithmetic). (3) Comparison between Rest 2 and Cognitive Task 2 (Sudoku). (4) Comparison between Rest 1 and Rest 2. (5) Comparison between Cognitive Task 1 and Cognitive Task 2. Upper 4 indices: The indices included in the time-domain analysis. Middle 5 indices: The indices included in the frequency-domain analysis. Lower 6 indices: The indices included in the chaos/complexity analysis. Emphasis indicates significance and direction: italics for significant increases and bold for significant decreases. The number of data points (sample size) was 27.

Index	(1) Rest 1 $\rightarrow$ Standing			(2) Rest 1 $\rightarrow$ Cognitive Task 1			(3) Rest 2 $\rightarrow$ Cognitive Task 2			(4) Rest 1 $\rightarrow$ Rest 2			(5) Cognitive Task 1 $\rightarrow$ Cognitive Task 2		
	p-value	Significant	Direction of change	p-value	Significant	Direction of change	p-value	Significant	Direction of change	p-value	Significant	Direction of change	p-value	Significant	Direction of change
**Time-domain analysis**															
SDNN	0.0593		$\downarrow$	**2.26E-04**	*	$\downarrow$	0.0697		$\downarrow$	0.355		$\uparrow$	6.39E-03		$\uparrow$
SDSD	**1.70E-04**	*	$\downarrow$	0.331		$\downarrow$	0.264		$\uparrow$	0.728		$\uparrow$	0.153		$\uparrow$
RMSSD	**1.70E-04**	*	$\downarrow$	0.331		$\downarrow$	0.264		$\uparrow$	0.728		$\uparrow$	0.153		$\uparrow$
pNN50	**6.65E-06**	*	$\downarrow$	0.657		$\downarrow$	0.355		$\uparrow$	0.952		$\downarrow$	0.153		$\uparrow$
**Frequency-domain analysis**															
LF	0.838		$\uparrow$	5.52E-03		$\downarrow$	0.801		$\uparrow$	0.307		$\uparrow$	*2.06E-04*	*	$\uparrow$
HF	**3.60E-04**	﻿*	$\downarrow$	0.0204		$\downarrow$	0.971		$\uparrow$	0.857		$\downarrow$	0.0355		$\uparrow$
LF/HF	*1.04E-05*	*	$\uparrow$	0.199		$\downarrow$	0.0903		$\downarrow$	0.407		$\uparrow$	0.307		$\uparrow$
LFnorm	*2.48E-05*	*	$\uparrow$	0.524		$\downarrow$	0.394		$\downarrow$	0.368		$\uparrow$	0.343		$\uparrow$
HFnorm	**2.48E-05**	*	$\downarrow$	0.524		$\uparrow$	0.394		$\uparrow$	0.368		$\downarrow$	0.343		$\downarrow$
**Chaotic/complexity analysis**															
ApEn	**5.16E-05**	*	$\downarrow$	*4.73E-04*	*	$\uparrow$	*1.27E-04*	*	$\uparrow$	0.153		$\downarrow$	0.0263		$\downarrow$
SampEn	**2.00E-05**	*	$\downarrow$	*6.75E-04*	*	$\uparrow$	*8.04E-04*	*	$\uparrow$	0.121		$\downarrow$	0.0105		$\downarrow$
Fractal Dimension	**8.33E-06**	*	$\downarrow$	*3.95E-04*	*	$\uparrow$	*9.55E-04*	*	$\uparrow$	0.274		$\downarrow$	0.0335		$\downarrow$
SD1/SD2	**4.32E-04**	*	$\downarrow$	*9.55E-04*	*	$\uparrow$	*2.37E-03*	*	$\uparrow$	0.838		$\downarrow$	0.464		$\downarrow$
CD	**1.04E-05**	*	$\downarrow$	*4.73E-04*	*	$\uparrow$	*1.70E-04*	*	$\uparrow$	0.254		$\downarrow$	0.0735		$\downarrow$
ICD	**7.44E-06**	*	$\downarrow$	*3.95E-04*	*	$\uparrow$	*1.70E-04*	*	$\uparrow$	0.254		$\downarrow$	0.0815		$\downarrow$

In comparison between (4) Rest 1 and Rest 2, no significant differences were observed in any HRV indices. In comparison between (5) Cognitive Task 1 and Cognitive Task 2, a significant difference was found only in LF, with no notable differences in the other indices. Both comparisons (4) and (5) therefore served as cross-validations of Experiments 1 and 2. Consequently, no substantial difference was found between the two types of cognitive tasks, namely mental arithmetic and Sudoku.

As an a priori power analysis could not be performed, post hoc power analyses were undertaken to assess the adequacy of the sample size. For all HRV indices, the corresponding p-values, standardised test statistics (*Z*) derived from the Wilcoxon signed-rank test, rank-based effect sizes ($r = \frac{Z}{\sqrt{N}}$, where *N* is the sample size), difference-based effect sizes (Cohen’s *d*; $d = \frac{\bar{D}}{s_D}$, where $\bar{D}$ is the mean of paired differences *D*, and $s_D$ is the standerd deviation of *D*), and achieved power ($1-\beta$) for each comparison are summarised in Table S17. Calculations for *d* and achieved power are approximate, assuming normality of the paired differences. For comparisons (1), (2), and (3), which aimed to detect significant differences, the observed effects demonstrated sufficient power, typically exceeding 0.8, at a sample size of $N = 27$ and a significance level of $\alpha = 0.01$ (i.e., before adjustment by the Holm-Bonferroni method). Under these conditions, the effect size detectable with a power of 0.8 was $d = 0.72$. For comparisons (4) and (5), intended to confirm similarity, the sensitivity to detect smaller differences was limited; such differences may not have been detected, and the results should therefore be interpreted with caution.

### Poincaré plot

**Fig. 3:**
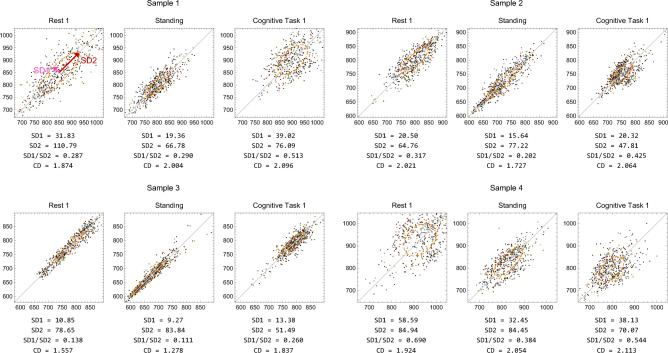
Typical examples of RRI Poincaré plots in Rest 1, Standing, and Cognitive Task 1. The Poincaré plot is a return map that plots points at the positions (*x*-axis, *y*-axis) = $(u_1, u_2), (u_2, u_3), (u_3, u_4),\cdots$ where $\{u_1, u_2, u_3, u_4...\}$ are time series RRI data. Let us look at the ellipse with orange dotted line. SD1 is the standard deviation along the minor axis. SD2 is the standard deviation along the major axis. An increase in the thickness of the Poincaré plot in the minor axis direction, that is, an increase in SD1/SD2 means that the change from the current RRI value $u_i$ to the next value $u_{i+1}$ becomes more widespread and leads to an uncertainty increase in the future. As a result, the chaos degree (CD), which is defined by conditional entropy, also increases. The elliptical shape of the Poincaré plot becomes more elongated in Standing than in Rest and becomes closer to a perfect circle in Cognitive Task than in Rest.

To further explore the characteristic changes in the chaos/complexity indices during the Standing and Cognitive Task, typical examples of Poincaré plots derived from the present experimental data are presented in Fig. 3. The Poincaré plot is a useful tool for visualising the relationships between data points in time-series data and for investigating the properties of the underlying dynamical system. It is particularly relevant to the SD1/SD2, CD, and ICD indices in the context of this study. As shown in Fig. 3, the elliptical distribution of the RRI data tended to collapse along the minor axis during the Standing condition compared to Rest, while the width along the minor axis increased during the Cognitive Task. These observations may reflect changes in the dynamics of HRV, associated with the decrease in chaos/complexity indices during Standing and their increase during the Cognitive Task.

### Chaos indicator ratio (CIR)

**Fig. 4:**
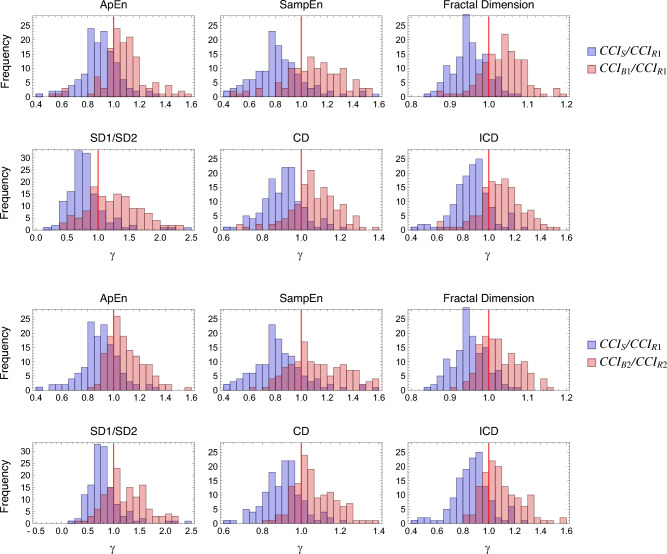
Histogram of the chaos indicator ratio (CIR) using 6 indices included in the chaos/complexity analysis. Upper: CIR of Rest to Standing (blue) and Rest to Cognitive Task 1 (red). Lower: CIR of Rest to Standing (blue) and Rest to Cognitive Task 2 (red). CCI means any of the six indices included in the chaos/complexity analysis.

The observed opposite directional changes in the chaos/complexity indices between physical and mental tasks, as described above, represent a novel feature not typically seen in conventional indices. This distinctive behaviour suggests the potential for using these indices to differentiate between the two conditions. To explore this further, let the chaos/complexity index (CCI: ApEn, SampEn, fractal dimension, SD1/SD2, CD, and ICD) in one condition be denoted as $CCI_1$, and in another condition as $CCI_2$, with their ratio,

1 $$\begin{aligned} \gamma = \frac{CCI_2}{CCI_1}, \end{aligned}$$

defined as the chaos indicator ratio (CIR). Fig. 4 presents a histogram of CIR, with $CCI_1$ representing the CCI for the Rest condition and $CCI_2$ representing the CCI for the Standing and Cognitive Task conditions, based on the experimental data. As shown in Fig. 4, the distributions of the physical and mental tasks are separated around $\gamma = 1$. This indicates that the CIR may serve as an universal measure for distinguishing between the two conditions based on the RRI data.

## Discussion

Although linear analysis of heart rate variability (HRV) has been standardised, nonlinear methods remain unstandardised, and their evaluation and interpretation are not yet clearly established. In this study, a comprehensive comparison of conventional autonomic indices and chaos/complexity indices during physical and mental tasks demonstrated markedly different patterns of change. Notably, the influence of brain activity on HRV was not reflected in conventional indices, whereas the chaos/complexity indices effectively captured these effects. These findings suggest that the chaotic and complex nature of HRV may be fundamental to understanding the interaction between the brain and the heart.

In the following, we review the key findings of this study and discuss each in turn. First, under the Standing condition, conventional autonomic indices indicated increased sympathetic activity and reduced parasympathetic activity. This observation is consistent with previous studies assessing autonomic function^1,26,27^.

In the Standing condition, almost all conventional and chaos/complexity indices exhibited significant changes. The conventional linear indices reflected the expected effects of orthostatic stress, namely baroreflex-mediated suppression of parasympathetic activity and activation of sympathetic activity, as widely reported in studies using head-up tilt. By contrast, all chaos/complexity indices showed a significant reduction. This reduction is consistent with earlier findings^18^, which demonstrated a marked decrease in the conditional entropy of RRI during head-up tilt. In that study, the decline in entropy was attributed to the predominance of a single periodic component, driven by an increase in the Mayer wave (a low-frequency oscillation linked to blood pressure variability) and a decrease in the respiratory component. Such changes reflect reduced parasympathetic activity and/or increased sympathetic activity^26,28^, leading to a simplification of RRI variability and, consequently, reduced entropy. Similar interpretations have been proposed in previous work showing that sympathetic activation and vagal withdrawal induced by postural change reduce HRV complexity^9,29^. In the present study, the same pattern was observed: the increase in the Mayer wave component and the reduction in the respiration-related component were reflected in a higher LF/HF ratio, increased LFnorm, and decreased HFnorm. These results therefore support the view that the physiological mechanisms underlying entropy reduction in RRI also account for the changes observed in the chaos/complexity indices.

During the Cognitive Task condition, all chaos/complexity indices showed significant increases. In contrast, no notable changes were observed in the periodic components (LF/HF, LFnorm, HFnorm) typically associated with sympathetic and parasympathetic nervous system activity. How, then, can these findings be reasonably explained? To interpret the experimental results, we first consider the influence of cognitive brain activity on HRV within the framework of the neurovisceral integration (NVI) model and insights from studies of brain networks. The NVI model and the principal large-scale brain networks are summarised in the following.

Historically, the hypothalamus was considered the central structure of the autonomic nervous system. However, recent advances in neuroscience have identified a broader network, referred to as the central autonomic network (CAN), which comprises the hypothalamus, anterior cingulate gyrus, insular cortex, and amygdala^30^. In addition, the neurovisceral integration (NVI) model^31^ has been proposed to describe how activation of the prefrontal cortex, through its connections with the CAN, influences cardiac regulation via autonomic pathways. An extended version of this model has also been developed, incorporating perspectives from functional and neuroanatomy^32^. That is, these models suggest that HRV is modulated by higher-order brain functions.

The three principal large-scale brain networks are the default mode network (DMN), which is associated with resting-state processes such as self-reflection and autobiographical memory; the central executive network (CEN), which is involved in higher-order cognitive functions including planning, decision-making, and working memory; and the salience network (SN), which detects the salience of internal and external stimuli and facilitates switching between networks^33^. The characteristics of these networks during cognitive tasks are summarised below. It is well established that the DMN is deactivated during cognitive tasks due to task-induced deactivation resulting from resource reallocation^34^. Recent studies have also indicated that the DMN and CEN may cooperate depending on the nature of the task^35^. The SN and CEN exhibit increased activity during cognitive processing^33^, with the SN playing a role in modulating and coordinating the activity of both the DMN and CEN^36,37^.

**Fig. 5:**
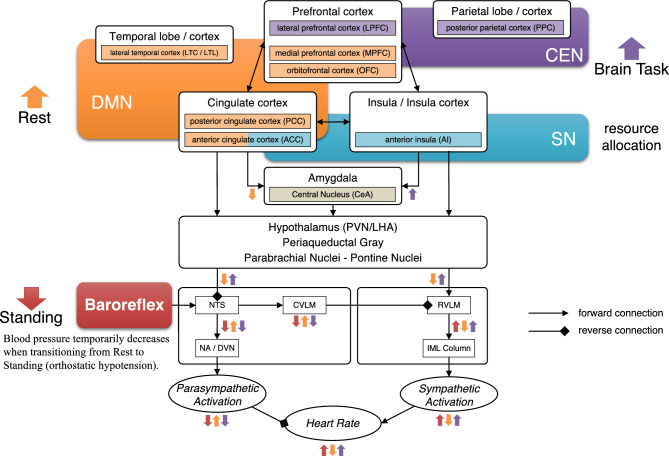
Expanded neurovisceral integration model incorporating findings from large-scale brain networks and fMRI studies. This schematic, drawn by the authors, illustrates an expanded version of the original neurovisceral integration model^31^. This model suggests that DMN activation (Rest) leads to deactivation of sympathetic nerve activity and activation of parasympathetic nerve activity. Conversely, CEN and SN activation (Cognitive Task) lead to activation of sympathetic nerve activity and deactivation of parasympathetic nerve activity. However, in the present experiment, these predicted changes in autonomic nerve activity were not significantly detected using conventional HRV analysis. Therefore, the chaoticity of HRV is presumed to reflect mechanisms other than a simple activation/deactivation pattern of the autonomic nervous system.

By incorporating the three network states observed during cognitive tasks into the original NVI model, a simple extension of the model can be proposed (Fig. 5). While this extended model accounts for the findings observed under Rest and Standing conditions, it does not explain the absence of significant changes in conventional frequency-domain indices during the Cognitive Task condition. These results therefore suggest the involvement of a chaotic mechanism that lies beyond the explanatory scope of the NVI model, and which may underlie the observed increase in the chaotic properties of HRV during cognitive tasks.

**Fig. 6:**
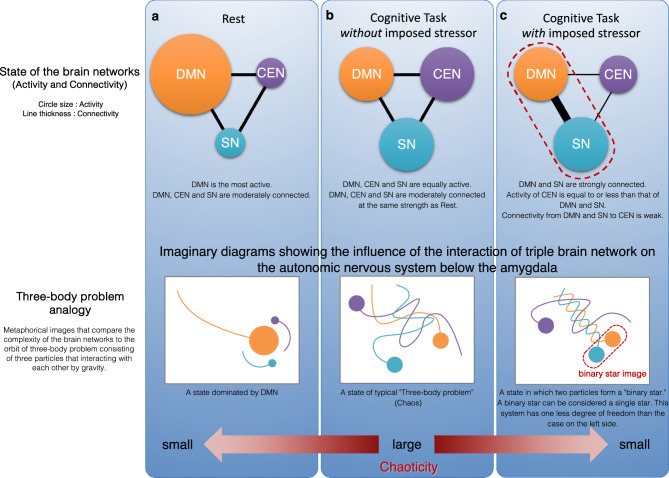
A conceptual framework that explains that higher-order brain function brings chaos to HRV. **(a)** In the Rest condition, the default mode network (DMN) is the most active. In addition, the DMN, central executive network (CEN) and salience network (SN) are moderately connected. Therefore, since the activity of the higher-order brain system, which consists of the DMN, CEN and SN, is controlled by one strong mode (DMN), the chaoticity in the system is small or does not appear. **(b)** In the Cognitive Task condition without imposed stressor, the DMN, CEN and SN are equally active. In addition, the DMN, CEN and SN are connected at the same strength as in Rest. That is, the higher-order brain system has three equal powered modes and is in a state of antagonism. In this case, the strong chaos may be caused in the system. According to the neurovisceral integration model, it can be explained that the chaos caused in the higher-order brain system brings chaos in autonomic nervous system, and finally, HRV becomes chaotic. **(c)** In the Cognitive Task condition with imposed stressor, the DMN, CEN and SN are equally active. However, the DMN and SN are strongly connected, and the connection to the CEN is weak. Therefore, the activity of the higher-order brain system is biased towards the DMN and SN, which are strongly connected. Then, the chaoticity in the system is smaller than that in Cognitive Task without imposed stressor.

We now turn to a more detailed consideration of why the chaotic nature of HRV increases during cognitive tasks. From the perspective of brain network interactions, it has been reported that the DMN and CEN may cooperate depending on task demands^35^, and that the DMN, CEN, and SN are functionally independent yet interconnected during cognitive processing. Furthermore, studies employing functional connectivity analysis of functional magnetic resonance imaging (fMRI) data have demonstrated that, under cognitive tasks involving stressors, connectivity between the DMN and SN increases, while connectivity between the DMN and CEN decreases^38,39^. By synthesising these findings within the conceptual framework of chaos theory, we tentatively propose the following hypothesis (Fig. 6): the complex and dynamic interactions among the DMN, CEN, and SN, which are three major large-scale brain networks, may give rise to chaotic fluctuations in HRV. This phenomenon may be understood by analogy with the three-body problem in physics, a canonical example of deterministic chaos. In that context, although the gravitational interactions among three bodies follow simple deterministic rules, their resulting trajectories become highly complex and unpredictable. Analogously, when the three brain networks operate at comparable levels of activity and connectivity, their mutual interactions may become sufficiently intricate to produce chaotic patterns in HRV. This hypothesis offers a plausible explanation for the significant increase in HRV chaos indices observed during the Cognitive Task condition, which could reflect a higher degree of interactional complexity facilitated by more balanced levels of network activity. Conversely, when there is a marked imbalance in either activity or connectivity among the networks, the system’s effective degrees of freedom may be reduced, thereby constraining the expression of chaos in HRV. This interpretation is consistent with the established understanding that chaos typically requires at least three interacting variables, or degrees of freedom, in continuous dynamical systems. Under the Rest condition, heightened activity in the DMN might induce such an imbalance, leading to a corresponding reduction in HRV complexity. Moreover, this hypothetical framework may also account for the decrease in HRV complexity observed during cognitive tasks under stressor-added conditions^19^, which could arise from a loss of degrees of freedom among the networks, particularly due to increased DMN-SN connectivity. It should be emphasised, however, that this explanation remains speculative and requires direct empirical testing.

One of the distinctive features of this study is the introduction of the chaos degree (CD) and improved chaos degree (ICD) as measures of the chaotic nature of HRV. These indices exhibited similar patterns to those observed in other nonlinear metrics, characterised by increases during the mental task and decreases during the physical task. Although the results do not indicate any substantial differences between the CD, ICD, and existing nonlinear indicators in terms of their responsiveness, the CD and ICD require considerably less computational effort. Their complexity is generally lower than that of ApEn and SampEn, as it depends on the number of divisions of the value range rather than on the length of the data series. Consequently, the CD and ICD may be particularly well-suited to applications that require real-time analysis or must operate under constrained computational resources.

However, aside from the RRI, no additional physiological data, such as respiration or electroencephalography (EEG), were collected in this study. Investigating the relationship between the observed increase in HRV chaos or complexity during mental tasks and concurrent changes in respiration and EEG signals remains an important direction for future research. Furthermore, the present experiment focused on changes in index values over a relatively short time scale of several minutes. Additional studies are required to elucidate the relationship between the chaotic characteristics of HRV and factors such as diminished concentration, fatigue, or mental health conditions over longer time scales.

## Limitations

This study has several limitations that should be acknowledged. First, participant recruitment was conducted at a single site, resulting in a relatively small and demographically skewed sample, predominantly comprising younger male participants. This lack of diversity constrains the generalisability of the findings to wider populations. Future research would benefit from multi-centre studies with larger and more heterogeneous cohorts to confirm and extend these results.

Second, factors such as medication use, caffeine intake, and arousal control were not strictly regulated. These uncontrolled variables may have influenced the outcomes. Nevertheless, to mitigate day-to-day fluctuations and increase reliability, we employed a protocol of five repeated measurements on different days.

Third, no specific instructions were provided to participants regarding whether to keep their eyes open or closed in each condition. Consequently, variations in visual input and sensory processing may have introduced additional variability in autonomic responses and HRV metrics. This uncontrolled factor represents a potential confound and should be carefully standardised in future investigations.

Fourth, this study did not include direct measurement of brain activity. The conclusions presented are based exclusively on heart rate variability (HRV) analyses. Although the experimental tasks, mental arithmetic and Sudoku, are recognized as activities that engage cognitive processes and brain activity, inferences about neural mechanisms remain indirect. Incorporating multimodal approaches, such as neuroimaging alongside HRV, would provide a more comprehensive understanding.

Fifth, although the Chaos indicator ratio (CIR) has a simple ratio-based structure, its formal mathematical properties have yet to be systematically characterised. While the use of ratios may reduce inter-individual variability, the independence of the CIR from established HRV indices and other conventional physiological measures remains unverified. The present findings indicate that the CIR can distinguish between physical and mental task conditions under experimental settings; however, its clinical interpretability and generalisability to real-world contexts have yet to be established. These considerations highlight important avenues for future research.

Finally, the discussion in this paper includes hypotheses and interpretative inferences that have not been empirically validated. Nonetheless, we contend that presenting ideas logically grounded in experimental results and previous studies can stimulate new research directions and advance scientific understanding in this field. Accordingly, the exploratory nature of this work should be viewed as a meaningful step toward future investigation rather than as definitive evidence.

## Conclusion

In this study, we observed a characteristic pattern in which the indices of chaos/complexity decreased during physical tasks and increased during mental tasks, based on a comprehensive analysis of RRI using multiple analytical approaches. These findings suggest that the chaos/complexity indices effectively capture features of HRV during mental tasks that are not reflected in conventional time-domain or frequency-domain measures. To aid in the interpretation of these experimental results, particularly the observed increase in HRV chaos during mental arithmetic and Sudoku, we outlined a tentative hypothesis linking brain network dynamics with the chaotic nature of HRV. This conceptual framework appears broadly consistent with existing literature on the neurovisceral integration model and large-scale brain networks. Although speculative, it may represent a promising avenue for future investigation into the mechanisms underlying HRV chaos and its potential applications. Furthermore, from an applied perspective, we introduced the CIR, which utilises chaos/complexity indices to differentiate between mental and physical task conditions.

The findings of this study suggest that the chaotic nature of HRV may reflect the influence of brain activity. The ability to infer brain states from RRI data holds considerable potential for both technological and clinical applications. Given that RRI can be acquired using compact heart rate sensors, its analysis offers a more accessible and cost-effective option compared with conventional techniques such as EEG or fMRI. Furthermore, the CD and ICD, newly introduced in this study for HRV analysis, exhibit robust performance in quantifying chaos while requiring minimal computational resources. This research is anticipated to contribute to the development of technologies capable of real-time assessment of physiological and psychological states, including fatigue, drowsiness, and stress.

Chaos/complexity analysis of RRI data offers novel insights that differ from those obtained through conventional methods for quantifying autonomic nervous system activity. Looking ahead, the application of chaos/complexity analysis to ECG data, which encompass even more physiological information than RRI, is likely to become increasingly important. While such analysis holds promise for addressing unresolved challenges, including the early detection of sudden cardiac death (SCD) and epileptic seizures, it should be emphasised that current findings are associative, and no causal relationship between chaos/complexity analysis of RRI (or ECG) data and these clinical outcomes has yet been established. Accordingly, further longitudinal and multifaceted investigations will be essential before translation into clinical practice can be realised. Nevertheless, chaos/complexity analysis is expected to have broad applicability across the life sciences, neuroscience, and medical research.

## Methods

### Participants

This experiment was conducted with 27 healthy participants. There were 20 participants in their 20s, 3 in their 30s, 3 in their 50s, and 1 in their 60s; also, 23 were males and 4 were females. Participants were recruited at a single site, and their involvement in the experiment was entirely voluntary, with clear prior notification that declining to participate would entail no disadvantages. This experiment was approved by the Research Ethics Committee of the Graduate School of Informatics, Kyoto University (approval number: KUIS-EAR-2019-006) and was conducted according to the principles of the Declaration of Helsinki. Informed consent was obtained from all participants of the study. Informed consent was obtained for publication of the identifiable images from the relevant subject.

### Experimental protocol

The participants wore a chest strap heart rate sensor (Polar H10), capable of measuring R-R intervals (RRIs). The Polar H10 has been extensively validated against medical-grade electrocardiograms in previous investigations^40–42^, which consistently demonstrate a high level of agreement with electrocardiogram recordings, indicating that it provides accurate and reliable heart rate measurements. According to the manufacturer’s documentation for developers^43^, the Polar H10 calculates RRIs in 1/1024-second increments. Conversely, several prior studies^41,44^ describe the electrocardiogram sampling rate of Polar H10 as 1000 Hz. Participants then took part in two experiments during which their RRIs were recorded under the following conditions. 

- Rest: participants sit quietly in a chair without performing any tasks (Fig. 1b-1).

- Standing: participants stand upright without leaning on any support (Fig. 1b-2).

- Cognitive task: participants sit in a chair and engage in a cognitive task, such as mental arithmetic or solving a Sudoku puzzle (Fig. 1b-3).

 Participants were not given specific instructions regarding whether to keep their eyes open or closed during any of the experimental conditions.

In Experiment 1, mental arithmetic (Fig. 1c) was used for the cognitive task. Participants’ RRIs were measured during three conditions: at rest (Rest 1) for 7 min, while standing (Standing) for 7 min, and during mental arithmetic (Cognitive Task 1) for 7 min. A 5-min break was provided between each condition (Fig. 1a). Five sets of trials were conducted on different days, providing repeated measurements to control for day-to-day fluctuations.

In Experiment 2, Sudoku^45^ (Fig. 1d) was used for the cognitive task. RRIs were measured at rest (Rest 2) for 7 min and during Sudoku (Cognitive Task 2) for 7 min. A 5-min break was provided between each condition (Fig. 1a). Five sets of trials were conducted on different days, providing repeated measurements to control for day-to-day fluctuations.

Measurement data that contained outliers due to poor contact between the sensor and the skin or missing data caused by interruptions in Bluetooth communication were discarded and not used in the analysis. Poor contact was assessed both quantitatively and qualitatively: quantitatively, measurements were flagged if 1% or more of the RRI data fell outside the physiologically plausible range of 200–2000 ms, and qualitatively, the RRI time series was visually inspected. In such cases, measurements were repeated to ensure that each participant had five valid datasets.

Sudoku was adopted in Experiment 2 because the mental arithmetic task in Experiment 1 required participants to frequently write answers, which could introduce physical load from hand movements that may have affected HRV measurements. In contrast, Sudoku involves fewer writing movements. If the results differed significantly between Experiment 1 (mental arithmetic) and Experiment 2 (Sudoku), it would suggest that hand movements influenced the outcomes. However, the results from both tasks showed similar trends across all indices, indicating that hand movements did not have a significant effect (Table 2).

To avoid the influence of active autonomic nervous activity after meals, all measurements were carried out within restricted time periods, specifically 10:30–12:00 and 14:45–18:15. Participants were also instructed not to eat or smoke immediately before the experiment.

### HRV analysis

We comprehensively analysed the RRI data measured in the experiment by using 15 indices (analytical methods) (Table 1) selected from the time-domain analysis, frequency-domain analysis, and chaos/complexity analysis. The index values was calculated from 420 s of data.

#### Time-domain analysis and frequency-domain analysis

Time-domain and frequency-domain analyses have long been used in the fields of HRV analysis and autonomic nerve function evaluation^1^. Using indices included in their analyses, we could determine the activity state of the sympathetic nervous system and/or parasympathetic nervous system. Time-domain analysis evaluates the statistical properties of RRIs. Indices of SDSD, RMSSD, and pNN50 that belong to time-domain analysis represent the indices to measure parasympathetic nervous system activity. Frequency-domain analysis assesses the magnitude of power in a particular frequency domain of RRIs. LF and LFnorm are indicators that reflect both sympathetic and parasympathetic nervous system activity, while HF and HFnorm measure parasympathetic nervous system activity. LF/HF represents the balance between sympathetic and parasympathetic nervous activity.

It should be noted that the interpretation of frequency-domain indices is not without controversy. Concerns have been raised regarding oversimplified interpretations, whereby LF power is taken to reflect both sympathetic and parasympathetic activity, HF power is assumed to represent parasympathetic activity, and the LF/HF ratio is regarded as an index of autonomic balance. In particular, LF is strongly influenced by baroreflex function, while HF is also affected by respiratory dynamics. It has been argued that variations in the LF/HF ratio may be confounded by factors unrelated to the actual sympathetic-parasympathetic balance, calling into question its physiological validity as an indicator of autonomic regulation^46^. To maintain clarity and avoid excessive complexity in the present manuscript, interpretations of these indices will be restricted to basic and minimal descriptions. The discussion will instead emphasise the responsiveness of each index to physical and mental task conditions.

#### Chaos/complexity analysis

HRV analysis is an analysis of “fluctuations”, but conventional analysis captures only some of the characteristics of RRI fluctuations. Careful observation of the Poincaré plot of RRI (Fig. 3) reveals that the possible range of values for the next RRI is narrower as the previous RRI is farther from the mean value and wider as it is closer to the mean value. However, such irregularities and complexity of fluctuations cannot be measured by conventional analysis, as described below.

In the time-domain analysis of the conventional analysis^1^, indices that measure the statistical characteristics of the fluctuation of RRI based on the variance or the standard deviation are defined, but it is not quantified how the value of RRI changes step by step. On the other hand, frequency-domain analysis measures the power of periodic components of fluctuations. Aperiodic effects are interpreted as noise. In the case of white noise, a small amount of power appears over the entire frequency range. In the case of 1/f fluctuation, the power that is inversely proportional to the frequency f appears. However, in the frequency-domain analysis of the conventional analysis^1^, there are no indices showing white noise or 1/f fluctuation.

The chaos degree that belongs to the chaos/complexity analysis described later captures the characteristics of the fluctuations that appear in the Poincaré plot of RRI as “the uncertainty of the possible values of RRI”. In Fig. S1, it is shown that the indices of CD, ICD, ApEn, SampEn belonging to the chaos/complexity analysis described later are independent of the indices of SDNN, SDSD, RMSSD belonging to the time-domain analysis of the conventional analysis.

Chaos/complexity analysis has recently come to be used in the field of HRV analysis and is also the focus of this article. In this article, we defined chaos/complexity analysis based on the following 6 indices: approximate entropy (ApEn), sample entropy (SampEn), SD1/SD2, fractal dimension, chaos degree (CD), and improved chaos degree (ICD). Details of each index are provided later. The correspondence between chaos in HRV and physiology has not been clearly explained, but some researchers suggest a relation to mental stress. We expected that chaos in HRV would be associated with higher-order brain function in our experiments. In the field of mathematical science, the phenomenon that a simple system (a system with a small degree of freedom) causes random-like behaviour is called chaos. Today, chaos is known to exist in various mathematical models, such as natural phenomena, social phenomena, and economic systems. Therefore, the chaos phenomenon may be observed in vital data such as ECG and EEG scans. One of the main conditions for a system to cause chaos is that the system has a property called “sensitivity to initial conditions”, which is quantified using an index called the Lyapunov exponent. However, it is necessary to know the dynamical system (i.e., the difference equation of the system) to calculate the Lyapunov exponent. Methods for estimating the Lyapunov exponent from only the obtained time series data include the methods of Wolf et al.^47^, Rosenstein et al.^48^, and Kantz^49^. These methods have the problem that they require a large number of data for computation, so their estimates are not stable for low-frequency data such as RRI. Therefore, they are not included in the group of chaos indicators listed above in this study. On the other hand, recently, it has become clear that chaos is closely related to conditional entropy, which is defined in the field of information theory. In addition, a method for estimating the Lyapunov exponent via conditional entropy has been developed, which can be calculated using only given data and does not require information about the dynamical system. Details are shown in subsection of CD and ICD. Five of the six indices included in the chaos/complexity analysis (ApEn, SampEn, SD1/SD2, CD, ICD) behave similarly to the Lyapunov exponent (Fig. S2).

Approximate entropy (ApEn)^50,51^ and sample entropy (SampEn)^52,53^: When time series data contain repeating patterns, values are easier to predict than when such patterns do not exist. ApEn measures the frequency of patterns contained in time series data and quantifies these patterns by the amount of information provided. A large ApEn value means that the pattern is repeated infrequently and the data are complex. ApEn was developed as an improvement on Kolmogorov-Sinai (KS) entropy in order to properly measure the regularity rate in time series data and is used in medical field measurements, such as heart rate, and in financial field measurements. SampEn is a measure of complexity similar to ApEn and is a modified version of ApEn. Macroscopically (if we ignore minor differences), SampEn shows almost the same behaviour as ApEn. In this study, the values of parameters m and r for the calculation of ApEn and SampEn, as selected in many previous studies, were set to $m=2$ and $r=0.2\sigma$, where $\sigma$ is the standard deviation of the data.

Fractal dimension (Higuchi dimension)^54,55^: The fractal dimension is a statistical method to quantify complexity and is explained as being derived from self-similarity. As a property of the fractal dimension, its value does not always show an integer value, and a large value means that the data are complex. Some practical methods have been proposed. In this article, we used the Higuchi dimension.

SD1/SD2 ratio^56,57^: SD1/SD2 is explained using Poincaré plot shown in Fig. 3. That is, SD1 is the standard deviation along the minor axis, i.e., the thickness of the ellipse viewed from the direction of $y = x$ (major axis). SD2 is the standard deviation along the major axis, i.e., the thickness of the ellipse viewed from the direction perpendicular to $y = x$ (minor axis). In particular, a large SD2 value means that the heart rate variability (HRV) is large, that is, the uncertainty is large. Therefore, if SD2 is relatively larger than SD1, the indices of complexity are large. In addition, SD1 and SD2 are highly correlated with SDNN and SDSD, respectively, in the time-domain analysis.

Chaos degree (CD)^22,23^ and improved chaos degree (ICD)^24,25^: The Lyapunov exponent is commonly used as a measure of chaos. However, it is difficult to calculate Lyapunov exponents if equations of dynamical systems are not given. On the other hand, entropic chaos degree (CD)^22^ is proposed as another measure of chaos that can be directly calculated from data. The definition of CD in difference equations is as follows. We assume that the one dimensional difference equation is determined by a map $f:I \rightarrow I(\equiv [a,b] \in \mathbb {R}^1,\;a,b\in \mathbb {R})$, i.e.,$M+1$ length observed data $\{x_0,x_1,x_2,\cdots ,x_M\} (\equiv \{x_n\}_{n=0}^{M})$ is given by $x_{n+1}=f(x_n)\,(n=0,1,2,\cdots ,M-1)$ for $x_0 \in I$. $A=\{A_i\}$ be a finite partition of *I* such that

2 $$\begin{aligned} I = \bigcup _{i=1}^{N} A_i,\quad A_i \cap A_j = \phi \;(i \ne j). \end{aligned}$$

The probability distribution *p*(*i*) and the joint probability distribution *p*(*i*, *j*) are given as

3 $$\begin{aligned}&p(i) = \frac{\#\{ x_n \in A_i \;\vert \; n = 0,1,\cdots ,M-1\}}{M},\end{aligned}$$

4 $$\begin{aligned}&p(i,j) = \frac{\#\{ x_n \in A_i,\;x_{n+1} \in A_j \;\vert \; n = 0,1,\cdots ,M-1\}}{M}. \end{aligned}$$

Then, CD for $\{x_n\}_{n=0}^{M}$ is defined by

5 $$\begin{aligned} \text {CD} = \sum _{i=1}^N \sum _{j=1}^N p(i,j) \log \frac{p(i)}{p(i,j)} = -\sum _{i=1}^{N} p(i) \sum _{j=1}^N p(j|i) \log p(j|i). \end{aligned}$$

where the conditional probability *p*(*j*|*i*) is defined as $p(j|i)=p(i,j)/p(i)$.

There is a difference between the CD and the Lyapunov exponent due to the finite partition. The difference can be interpreted as the amount of information about how the output data $f(A_i)$ are distributed in each $A_j$. The ICD, which is obtained by subtracting the amount of information from the CD, is defined as follows. In addition to the definition of CD above, *q*(*i*, *j*) is defined as the ratio of $f(A_i)$, which is the area where $A_i$ is mapped by *f*, to $A_j$, that is,

6 $$\begin{aligned} q(i,j) = \frac{\Vert f(A_i) \cap A_j \Vert }{\Vert A_j \Vert }. \end{aligned}$$

ICD^24,25^ is defined by subtracting the average amount of information $-\log q(i,j)$ from CD as

7 $$\begin{aligned} \text {ICD} = -\sum _{i=1}^{N} p(i) \sum _{j=1}^N p(j|i) \{ \log p(j|i) - \log q(i,j) \}. \end{aligned}$$

Computation of *q*(*i*, *j*) using observed data $\{x_n\}_{n=0}^{M}$ is as follows. The component $A_j$ is divided into *Q* equipartition $B_{j,l}$ such that

8 $$\begin{aligned} A_j = \bigcup _{l=1}^{Q} B_{j,l},\quad B_{j,l} \cap B_{j,l'} = \phi \;(l \ne l'). \end{aligned}$$

Then,

9 $$\begin{aligned} q(i,j) = \frac{ \#\{ \;\#\{ x_{n} \in A_i,\,x_{n+1} \in B_{j,l} \;\vert \; n=0,1,2,\cdots ,M-1\} > 0 \;|\; l=1,2,\cdots ,Q\} }{Q}. \end{aligned}$$

In this study, *M* and *I* for the culcuration of CD is set to $M=20$ and $I=[\mu -2\sigma , \mu +2\sigma ]$, where $\mu$ is the average of the data and $\sigma$ is the standard deviation of the data, and *M*, *Q* and *I* for the culcuration of ICD is set to $M=10$, $Q=4$ and $I=[\mu -2\sigma , \mu +2\sigma ]$, where $\mu$ is the average of the data and $\sigma$ is the standard deviation of the data.

### Sample size and power analysis

The chaos degree (CD) and improved chaos degree (ICD) metrics introduced in this study represent the first application of these measures in HRV analysis. Because prior data describing variability were unavailable, it was not possible to conduct a reliable unified a priori power calculation. Consequently, the sample size was determined based on feasibility considerations. Post hoc power analyses for each metric are summarised in Table S17 to support transparency in the study’s statistical power assessment.

### Statistical significance test

The primary aim of this study was to examine differences between Rest and Standing outcomes, and between Rest and Cognitive Task outcomes. Statistical significance was assessed for the following five paired comparisons under the null hypothesis ($H_0$) that no difference exists between the two conditions, and the alternative hypothesis ($H_1$) that a difference does exist: (1) Rest 1 vs Standing, (2) Rest 1 vs Cognitive Task 1, (3) Rest 2 vs Cognitive Task 2, (4) Rest 1 vs Rest 2, and (5) Cognitive Task 1 vs Cognitive Task 2. Comparisons (1) and (2) correspond to Rest vs Standing and Rest vs Cognitive Task in Experiment 1, whereas comparison (3) corresponds to Rest vs Cognitive Task in Experiment 2. Comparisons (4) and (5) serve as cross-validation tests to confirm that no significant differences were present between equivalent conditions (Rest vs Rest; Cognitive Task vs Cognitive Task) across experiments. As normality could not be assumed for all heart rate variability (HRV) indices, p-values for each paired comparison were obtained using the Wilcoxon signed-rank test. The significance threshold ($\alpha$) was set at $1\%$. To account for multiple comparisons, the Holm-Bonferroni method was applied, thereby controlling the familywise error rate below $\alpha$.

P-values were computed using the SignedRankTest function in Wolfram Mathematica (version 14.3; https://www.wolfram.com/mathematica/). Post hoc power and sensitivity analyses were conducted with G*Power (version 3.1.9.7; https://www.psychologie.hhu.de/arbeitsgruppen/allgemeine-psychologie-und-arbeitspsychologie/gpower).

## Supplementary Information

Supplementary Information.

## Data Availability

The RRI data that support the findings of this study are openly available at https://doi.org/10.57723/285156 in KURENAI, Kyoto University Research Information Repository.
