# Correction: Chaotic fluctuations mark the sign of mental activity in task-based heart rate variability

**DOI:** 10.1038/s41598-026-50485-3

**Published:** 2026-04-28

**Authors:** Tomoyuki Mao, Hidetoshi Okutomi, Ken Umeno

**Affiliations:** 1 Department of Applied Mathematics and Physics, Graduate School of Informatics, Kyoto University, Sakyo-ku, Kyoto, 606-8501 Japan; 2 Technology Management Division, Toshiba Information Systems (Japan) Corporation, Kawasaki-ku, Kawasaki, Kanagawa 210-8540 Japan

Correction to: *Scientific Reports* 10.1038/s41598-026-43385-z, published online 24 March 2026

In the original version of this Article, author Ken Umeno was omitted as a corresponding author. Correspondence and requests for materials should also be addressed to umeno.ken.8z@kyoto-u.ac.jp.

The original Article has been corrected.

