# Supplementary material for: Chaotic fluctuations mark the sign of mental activity in task-based heart rate variability
Source: Sci Rep. 2026 Mar 24;16:9221. doi: 10.1038/s41598-026-43385-z (PMC13013681; doi:10.1038/s41598-026-43385-z)
Supplement: Supplementary file 1 — Supplementary Information. [file 41598_2026_43385_MOESM1_ESM.pdf]

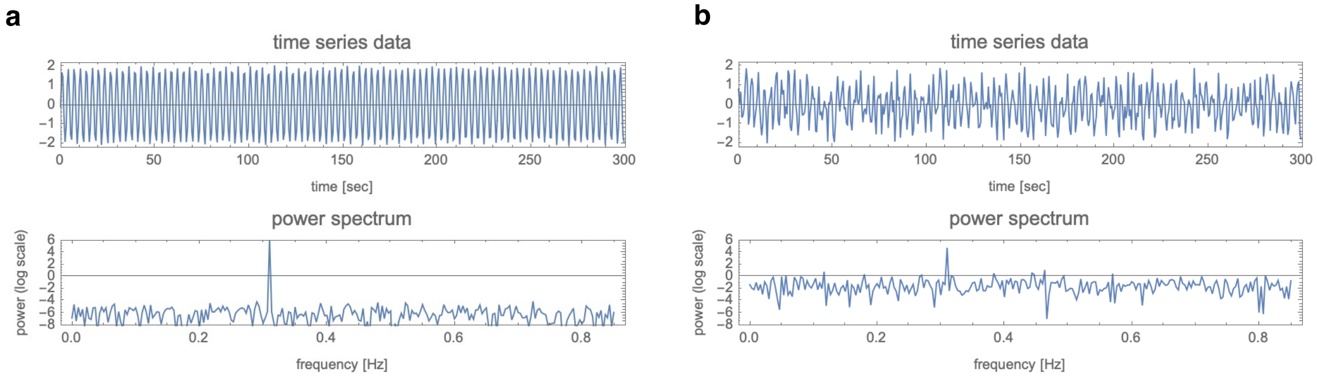

c

		a		b
time-domain analysis	SDNN	1.416	>	0.919
	SDSD	1.530	>	1.118
	RMSSD	1.530	>	1.118
chaos/complexity analysis	CD	1.286	<	2.067
	ICD	1.016	<	1.773
	ApEn	0.140	<	1.356
	SampEn	0.155	<	2.068

Figure S1. Independence of chaos/complexity analysis and conventional time-domain analysis. **(a)** A highly periodic wave generated by adding random numbers uniformly distributed in the interval $[-0.1, 0.1]$ to a sine wave with a frequency of 0.31 [Hz] and an amplitude of 2. **(b)** A highly irregular wave generated by adding random numbers uniformly distributed in the interval $[-1, 1]$ to a sine wave with a frequency of 0.31 [Hz] and an amplitude of 1. **(c)** SDNN, SDSD, RMSSD, CD, ICD, ApEn, SampEn of two time series data shown in (a) (b). Since time series data (a) is larger in amplitude and more periodic (less complex) than (b), SDNN, SDSD, and RMSSD take large values, while CD, ICD, ApEn, and SampEn are small. Conversely, time series data (b) has smaller amplitude and lower periodicity (higher complexity) than (a), so SDNN, SDSD, and RMSSD take small values, while CD, ICD, ApEn, and SampEn take large values. In other words, the chaos/complexity analysis indices (CD, ICD, ApEn, and SampEn) are independent of the time-domain analysis indices (SDNN, SDSD, and RMSSD) of the conventional analysis.

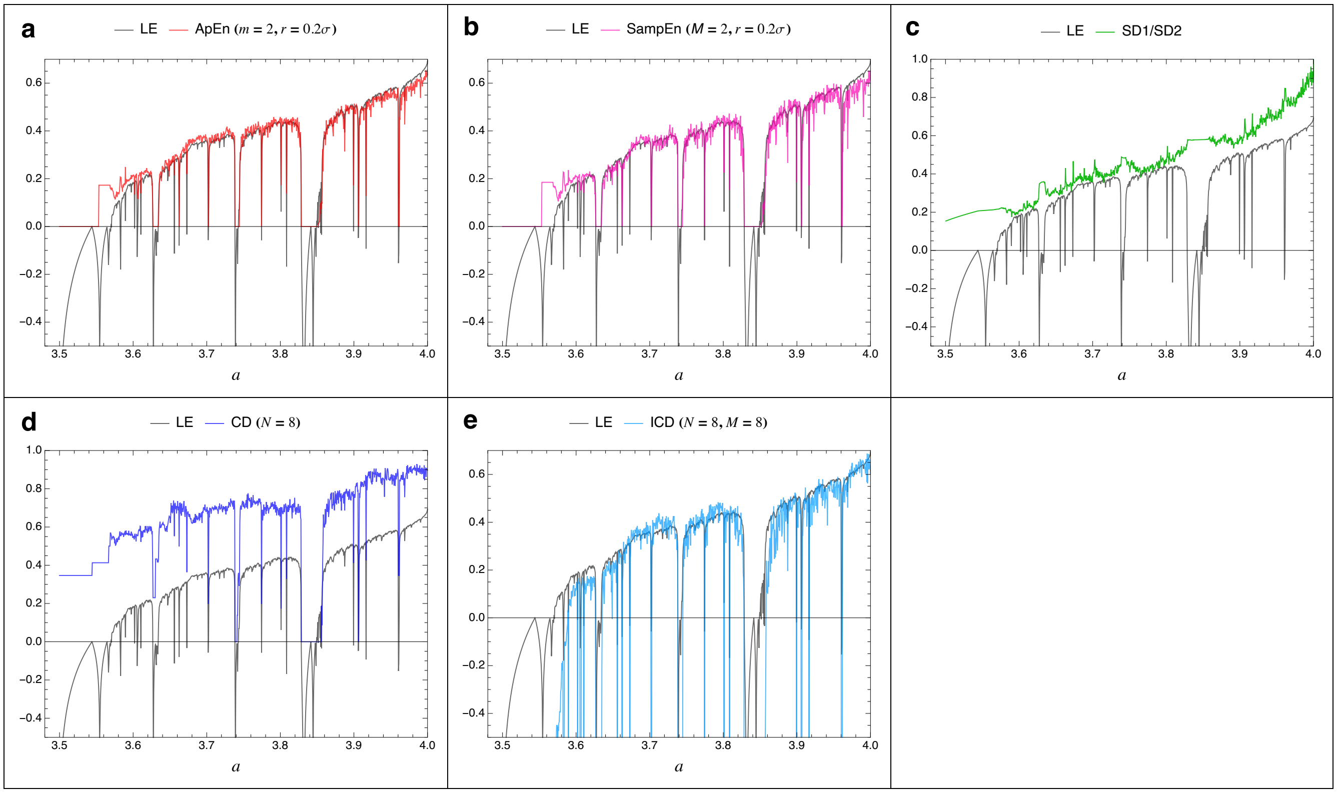

Figure S2. Comparison of Lyapunov exponent (LE) and chaos indices (ApEn, SampEn, SD1/SD2, CD, and ICD) of Logistic map. **(a)** ApEn and LE. **(b)** SampEn and LE. **(c)** SD1/SD2 and LE. **(d)** CD and LE. **(e)** ICD and LE. In each figure from (a) to (e), the black solid line shows LE of the logistic map, $x_{n+1} = ax_n(1-x_n)$, at its control parameter a. Intervals where LE is positive are chaos region, and the larger LE is, the stronger chaos. In contrast, intervals where LE is negative are nonchaos region (i.e., the periodic region). ApEn and SampEn give good approximations of LE, but the computational complexity is $O(n^2)$ for data size n. CD has a numerical difference from LE, but it behaves almost the same as LE. ICD has a value closer to LE. The computational complexity of CD and ICD is $O(n)$ for data size n. Therefore, it is much more advantageous to use CD or ICD.

	Experiment 1						Experiment 2			
	Rest 1		Standing		Cognitive Task 1		Rest 2		Cognitive Task 2	
	μ	σ	μ	σ	μ	σ	μ	σ	μ	σ
**time-domain analysis**										
SDNN	55.96	18.41	51.59	16.99	45.23	12.44	57.32	18.48	52.67	16.48
SDSD	32.47	14.85	24.50	10.86	31.94	13.17	32.50	14.05	37.37	18.63
RMSSD	32.47	14.85	24.50	10.86	31.94	13.17	32.50	14.05	37.37	18.63
pNN50	0.14	0.14	0.06	0.08	0.13	0.13	0.13	0.13	0.14	0.13
**frequency-domain analysis**										
LF	490.43	377.31	520.07	384.93	313.69	198.08	494.51	299.71	776.33	1657.57
HF	197.22	169.74	121.45	108.09	143.79	117.63	184.47	155.33	221.78	253.25
LF/HF	3.93	2.55	7.02	5.07	3.30	2.13	4.48	3.46	3.62	2.28
LFnorm	71.56	12.78	81.08	8.68	70.20	10.52	72.47	15.96	71.86	12.11
HFnorm	28.44	12.78	18.92	8.68	29.80	10.52	27.53	15.96	28.14	12.11
**chaotic/complexity analysis**										
ApEn	1.13	0.10	1.00	0.19	1.22	0.10	1.11	0.11	1.19	0.09
SampEn	1.31	0.23	1.06	0.27	1.49	0.23	1.26	0.24	1.40	0.20
Fractal Dimension	1.76	0.08	1.67	0.08	1.82	0.07	1.74	0.09	1.79	0.07
SD1/SD2	0.31	0.09	0.24	0.06	0.38	0.10	0.30	0.10	0.36	0.11
CD	1.94	0.17	1.74	0.22	2.08	0.18	1.92	0.18	2.03	0.15
ICD	1.34	0.16	1.16	0.19	1.48	0.16	1.33	0.17	1.43	0.14

Table S1. Index values as experimental results : Mean μ and standard deviation σ of index values in each state in Experiment 1 and 2. Upper 4 indices: The indices included in the time-domain analysis. Middle 5 indices: The indices included in the frequency-domain analysis. Lower 6 indices: The indices included in the chaotic/complexity analysis. The number of data points (sample size) was 27.

SDNN

Participant ID	Experiment 1						Experiment 2			
	Rest 1		Standing		Cognitive Task 1		Rest 2		Cognitive Task 2	
	μ	σ	μ	σ	μ	σ	μ	σ	μ	σ
1	83.03	27.93	45.33	7.41	64.81	16.81	70.19	8.30	65.96	8.37
2	44.97	14.25	40.59	14.71	44.42	5.72	47.15	5.87	59.56	12.44
3	28.84	7.25	25.84	2.75	22.46	3.52	31.56	5.54	28.12	7.18
4	31.89	6.82	42.51	10.29	33.52	1.78	50.23	12.57	36.69	2.61
5	87.80	10.16	92.13	24.65	60.48	8.71	97.66	18.16	80.25	10.14
6	41.60	10.45	34.88	7.65	39.21	6.62	46.69	13.35	49.95	18.48
7	45.69	7.19	41.13	7.95	32.69	2.61	48.12	6.37	82.53	87.44
8	24.26	5.10	32.77	15.53	21.11	4.07	25.31	6.82	21.33	3.58
9	43.73	6.56	53.36	15.13	36.17	13.48	61.06	11.49	42.76	6.48
10	49.52	4.79	43.96	6.09	43.60	5.02	50.34	10.04	48.89	11.46
11	59.23	6.11	46.77	8.82	62.17	22.41	66.91	36.46	68.31	16.73
12	30.07	2.30	24.29	3.64	26.43	5.29	30.22	3.54	33.41	5.95
13	76.53	12.76	53.48	14.75	53.38	3.26	57.00	22.16	41.78	14.31
14	63.05	8.79	86.79	22.06	52.40	3.61	57.17	10.80	43.25	10.15
15	61.16	8.51	64.39	16.27	66.54	7.24	61.48	20.03	84.23	16.46
16	80.07	17.37	55.04	10.89	45.12	2.65	93.95	12.09	58.56	14.24
17	52.71	9.59	50.63	8.77	35.85	5.53	54.13	8.54	34.81	5.68
18	35.93	4.29	31.41	4.98	45.86	6.85	44.12	13.39	40.44	5.56
19	82.36	11.33	72.78	9.81	48.94	7.09	89.07	8.79	59.53	6.04
20	62.27	17.33	61.08	13.01	49.38	14.40	66.66	28.31	54.29	6.07
21	52.68	7.61	42.53	6.31	36.59	5.61	64.86	21.47	61.64	9.30
22	64.69	22.99	60.92	11.46	46.39	6.61	61.58	22.97	67.05	37.61
23	51.97	14.54	53.05	13.75	43.45	10.40	31.32	8.53	35.11	5.50
24	49.42	6.27	41.53	9.35	40.86	3.73	70.00	14.72	56.15	7.91
25	87.81	5.70	75.37	16.30	58.93	3.07	76.62	8.66	67.52	6.82
26	63.11	2.43	56.22	12.80	49.22	7.70	50.78	3.58	51.84	5.12
27	56.53	12.64	64.14	8.27	61.23	6.79	43.48	8.88	48.06	8.59

Table S2. Within-subject means μ and standard deviations σ of five repeated measurements for SDNN in each experimental condition.

SDSD

Participant ID	Experiment 1						Experiment 2			
	Rest 1		Standing		Cognitive Task 1		Rest 2		Cognitive Task 2	
	μ	σ	μ	σ	μ	σ	μ	σ	μ	σ
1	45.14	8.99	17.57	3.59	43.20	6.39	49.89	7.99	47.19	9.21
2	27.91	19.64	24.42	24.65	27.76	7.50	25.10	3.37	38.74	12.53
3	18.35	9.33	9.71	3.16	11.74	2.90	19.71	5.66	14.79	4.28
4	14.63	5.64	13.10	2.36	13.80	1.51	23.19	7.63	20.50	5.60
5	60.34	13.47	49.16	14.82	41.68	18.78	71.57	9.21	63.53	8.51
6	27.91	6.18	17.66	4.45	32.02	4.87	32.01	11.21	37.69	17.53
7	25.49	3.65	13.88	6.05	26.27	4.98	24.11	3.06	90.99	137.03
8	9.85	4.29	14.69	20.49	9.93	2.28	8.87	3.95	10.38	2.92
9	17.62	4.83	37.00	32.02	32.68	23.32	33.26	5.99	18.66	3.98
10	22.28	2.98	16.80	2.13	24.21	5.05	22.52	4.62	24.81	7.33
11	21.56	4.22	13.65	2.70	47.24	41.59	24.39	15.07	39.77	8.59
12	22.61	10.93	13.88	1.64	20.73	6.72	24.92	4.61	35.98	12.87
13	42.67	11.13	29.35	11.10	39.60	3.54	29.39	14.89	24.42	11.28
14	64.35	16.95	45.09	12.40	53.55	5.87	48.94	9.83	47.07	11.52
15	39.34	7.39	26.83	3.11	56.04	7.98	35.08	11.68	69.06	23.81
16	39.01	4.86	36.86	13.81	40.02	4.52	38.14	5.25	43.49	4.59
17	23.13	3.41	24.05	8.94	20.31	5.37	22.59	4.33	19.42	3.98
18	15.96	4.26	12.53	2.35	12.40	2.49	17.56	4.37	19.23	4.00
19	34.00	4.02	25.25	5.06	32.33	5.82	33.22	4.03	37.32	3.50
20	41.25	15.53	31.04	9.48	31.00	11.18	58.82	54.16	30.67	4.79
21	22.04	6.75	15.29	3.09	23.86	5.73	29.99	5.20	43.68	14.97
22	33.66	12.23	28.45	17.10	28.47	8.19	37.12	18.20	63.51	62.86
23	37.72	12.22	26.84	7.84	30.47	8.92	15.16	4.97	17.32	4.20
24	20.15	2.81	15.10	2.81	22.78	5.77	33.51	9.72	30.08	5.88
25	58.69	10.34	41.15	8.43	51.70	6.32	43.20	5.49	44.72	6.32
26	54.80	6.64	33.16	4.51	39.47	4.61	50.17	10.12	44.84	8.84
27	36.13	6.25	28.99	5.06	49.24	6.47	24.96	4.82	31.03	6.40

Table S3. Within-subject means μ and standard deviations σ of five repeated measurements for SDSD in each experimental condition.

RMSSD

Participant ID	Experiment 1						Experiment 2			
	Rest 1		Standing		Cognitive Task 1		Rest 2		Cognitive Task 2	
	μ	σ	μ	σ	μ	σ	μ	σ	μ	σ
1	45.14	8.99	17.57	3.59	43.20	6.39	49.89	7.99	47.19	9.21
2	27.91	19.64	24.42	24.65	27.76	7.50	25.10	3.37	38.74	12.53
3	18.35	9.33	9.71	3.16	11.74	2.90	19.71	5.66	14.79	4.27
4	14.63	5.64	13.10	2.36	13.80	1.51	23.19	7.63	20.50	5.60
5	60.34	13.47	49.16	14.83	41.68	18.78	71.58	9.21	63.53	8.51
6	27.91	6.18	17.66	4.45	32.02	4.87	32.01	11.21	37.69	17.53
7	25.49	3.65	13.89	6.05	26.27	4.98	24.11	3.06	90.99	137.03
8	9.85	4.29	14.69	20.49	9.93	2.28	8.87	3.95	10.38	2.92
9	17.62	4.83	37.00	32.02	32.68	23.32	33.26	5.99	18.66	3.98
10	22.28	2.99	16.81	2.13	24.21	5.05	22.52	4.62	24.82	7.33
11	21.56	4.22	13.65	2.70	47.24	41.59	24.40	15.07	39.77	8.59
12	22.61	10.93	13.88	1.64	20.73	6.72	24.92	4.61	35.98	12.87
13	42.67	11.13	29.35	11.10	39.60	3.54	29.39	14.89	24.42	11.28
14	64.35	16.95	45.09	12.40	53.55	5.87	48.94	9.83	47.07	11.52
15	39.34	7.39	26.83	3.11	56.04	7.98	35.08	11.68	69.07	23.81
16	39.01	4.86	36.86	13.81	40.02	4.52	38.14	5.25	43.49	4.59
17	23.14	3.41	24.05	8.94	20.32	5.37	22.59	4.33	19.42	3.98
18	15.96	4.26	12.53	2.35	12.40	2.49	17.56	4.37	19.23	4.00
19	34.00	4.02	25.25	5.06	32.33	5.82	33.22	4.03	37.32	3.50
20	41.26	15.55	31.04	9.48	31.00	11.18	58.82	54.16	30.67	4.80
21	22.04	6.75	15.29	3.09	23.86	5.73	30.00	5.20	43.68	14.97
22	33.66	12.23	28.45	17.10	28.47	8.19	37.12	18.20	63.51	62.86
23	37.72	12.22	26.84	7.84	30.47	8.92	15.16	4.97	17.32	4.20
24	20.15	2.81	15.10	2.81	22.78	5.77	33.51	9.72	30.08	5.88
25	58.69	10.34	41.15	8.44	51.70	6.32	43.20	5.49	44.72	6.32
26	54.80	6.64	33.16	4.51	39.47	4.61	50.17	10.12	44.84	8.84
27	36.13	6.25	28.99	5.06	49.24	6.47	24.96	4.82	31.03	6.40

Table S4. Within-subject means μ and standard deviations σ of five repeated measurements for RMSSD in each experimental condition.

pNN50

Participant ID	Experiment 1						Experiment 2			
	**Rest 1**		**Standing**		**Cognitive Task 1**		**Rest 2**		**Cognitive Task 2**	
	μ	σ	μ	σ	μ	σ	μ	σ	μ	σ
1	0.2558	0.1022	0.0093	0.0049	0.2220	0.0644	0.3094	0.0820	0.2760	0.1082
2	0.0200	0.0159	0.0101	0.0116	0.0790	0.0704	0.0412	0.0238	0.0998	0.0517
3	0.0335	0.0573	0.0003	0.0006	0.0006	0.0013	0.0174	0.0169	0.0045	0.0042
4	0.0121	0.0196	0.0049	0.0019	0.0032	0.0024	0.0355	0.0382	0.0221	0.0194
5	0.4383	0.1398	0.3001	0.1406	0.2065	0.1637	0.5301	0.0521	0.4881	0.0721
6	0.0820	0.0500	0.0146	0.0187	0.1190	0.0627	0.1352	0.1050	0.1663	0.1042
7	0.0630	0.0309	0.0125	0.0178	0.0455	0.0440	0.0535	0.0233	0.0504	0.0245
8	0.0000	0.0000	0.0008	0.0015	0.0003	0.0006	0.0030	0.0060	0.0015	0.0019
9	0.0221	0.0180	0.0125	0.0110	0.0202	0.0191	0.0651	0.0412	0.0211	0.0138
10	0.0309	0.0225	0.0115	0.0052	0.0359	0.0251	0.0410	0.0292	0.0600	0.0569
11	0.0282	0.0163	0.0071	0.0045	0.0937	0.0555	0.0582	0.0873	0.1833	0.0783
12	0.0029	0.0059	0.0004	0.0008	0.0094	0.0187	0.0281	0.0306	0.0421	0.0317
13	0.2115	0.0990	0.0607	0.0363	0.2075	0.0577	0.1048	0.0857	0.0776	0.0803
14	0.4311	0.1561	0.2356	0.1069	0.4037	0.0812	0.3208	0.0941	0.3193	0.1358
15	0.2137	0.0892	0.0553	0.0254	0.3973	0.0736	0.1606	0.1420	0.3496	0.1499
16	0.2019	0.0674	0.0853	0.0492	0.2397	0.0736	0.1720	0.0588	0.2687	0.0468
17	0.0378	0.0285	0.0257	0.0216	0.0315	0.0361	0.0258	0.0165	0.0349	0.0228
18	0.0098	0.0131	0.0028	0.0018	0.0041	0.0032	0.0165	0.0132	0.0205	0.0175
19	0.1356	0.0373	0.0627	0.0382	0.1325	0.0637	0.1352	0.0401	0.1925	0.0523
20	0.1430	0.0987	0.0633	0.0379	0.0970	0.0735	0.1443	0.0850	0.1127	0.0512
21	0.0376	0.0447	0.0056	0.0059	0.0416	0.0502	0.0978	0.0596	0.1257	0.1047
22	0.1429	0.1011	0.0371	0.0392	0.0889	0.0791	0.1918	0.1789	0.1988	0.1739
23	0.1889	0.1416	0.0759	0.0699	0.1185	0.0888	0.0109	0.0148	0.0169	0.0167
24	0.0162	0.0113	0.0073	0.0106	0.0250	0.0193	0.1333	0.0794	0.0835	0.0353
25	0.3471	0.0680	0.1906	0.0770	0.3712	0.0734	0.2370	0.0477	0.2648	0.0712
26	0.4290	0.0811	0.1345	0.0531	0.1917	0.0757	0.3855	0.1471	0.2811	0.1205
27	0.1716	0.0732	0.0884	0.0449	0.3390	0.0661	0.0619	0.0392	0.1236	0.0650

Table S5. Within-subject means μ and standard deviations σ of five repeated measurements for pNN50 in each experimental condition.

LF

Participant ID	Experiment 1						Experiment 2			
	**Rest 1**		**Standing**		**Cognitive Task 1**		**Rest 2**		**Cognitive Task 2**	
	μ	σ	μ	σ	μ	σ	μ	σ	μ	σ
1	425.50	83.44	237.28	47.09	362.61	131.21	497.39	100.78	475.77	79.45
2	427.24	222.31	377.85	252.62	389.31	130.64	478.78	261.51	644.76	183.71
3	69.63	26.52	34.58	3.34	38.72	10.11	59.30	24.30	53.52	16.74
4	205.17	59.14	378.88	68.86	251.23	50.81	452.01	171.51	288.90	58.66
5	1008.22	218.51	1095.00	260.93	370.67	155.09	1035.64	480.50	715.19	115.79
6	251.42	115.77	219.62	86.06	147.58	38.03	260.34	140.91	324.53	154.62
7	578.52	283.08	248.44	122.88	126.32	13.04	568.92	298.98	8927.17	17512.37
8	65.04	19.45	175.12	167.74	86.50	32.78	83.73	30.21	94.25	38.42
9	344.94	122.51	1026.20	1095.41	332.17	363.01	467.81	152.95	430.50	144.16
10	495.86	146.63	485.60	72.78	376.89	84.47	701.96	245.87	520.94	221.04
11	427.02	182.04	445.33	158.62	916.96	733.83	543.00	253.60	813.72	326.20
12	63.23	10.82	46.79	14.36	27.11	7.78	74.10	29.73	59.32	12.47
13	1584.31	628.13	742.75	315.92	485.42	204.20	1014.58	655.59	445.24	247.46
14	364.51	145.45	1625.46	1819.17	253.95	84.20	191.22	73.81	225.32	125.23
15	446.56	89.66	618.26	193.58	746.61	273.64	421.91	263.12	1402.91	596.69
16	721.94	259.52	466.78	125.08	317.80	31.05	858.82	293.86	429.93	119.08
17	262.41	44.80	224.27	38.64	90.63	26.79	241.13	57.55	139.34	59.95
18	155.91	41.74	161.94	78.30	275.31	110.52	355.75	199.81	393.39	163.22
19	1149.09	309.84	954.80	211.82	365.59	88.05	1235.27	277.54	425.72	42.37
20	692.86	284.67	567.31	159.11	302.73	188.49	701.89	684.52	400.16	52.16
21	187.47	88.02	181.44	52.02	182.09	84.18	466.76	190.50	562.14	179.56
22	566.97	278.30	594.68	203.88	320.40	84.89	624.19	304.98	1205.23	1657.90
23	434.90	247.71	553.36	292.62	318.33	132.99	232.01	89.62	322.68	71.37
24	233.24	40.01	131.56	30.01	164.10	27.81	520.30	236.96	273.41	36.38
25	1277.51	425.65	1109.08	647.89	478.03	94.92	701.83	204.19	605.35	153.51
26	356.06	47.12	526.30	314.23	280.13	66.25	244.89	29.66	368.07	64.52
27	446.04	153.10	813.31	293.44	462.30	124.64	318.15	100.10	413.55	156.36

Table S6. Within-subject means μ and standard deviations σ of five repeated measurements for LF in each experimental condition.

HF

Participant ID	Experiment 1						Experiment 2			
	Rest 1		Standing		Cognitive Task 1		Rest 2		Cognitive Task 2	
	μ	σ	μ	σ	μ	σ	μ	σ	μ	σ
1	293.05	88.14	75.34	21.93	198.65	59.07	328.17	83.34	289.77	99.16
2	178.76	237.52	142.11	221.68	103.55	43.77	82.99	28.16	183.24	110.26
3	115.94	96.08	27.56	18.71	36.98	17.29	83.69	41.71	50.13	22.25
4	41.94	29.57	29.35	9.24	31.36	8.20	80.79	55.88	61.08	26.27
5	460.06	89.20	318.78	159.44	113.56	74.13	538.54	173.46	348.02	77.91
6	133.87	49.01	54.38	25.12	103.37	28.26	165.48	85.88	234.17	128.81
7	113.90	13.74	43.67	28.93	78.15	24.09	103.03	33.39	1299.11	2425.33
8	19.95	13.49	90.62	167.26	22.13	8.68	16.69	11.89	25.56	11.46
9	63.94	28.47	413.36	666.09	215.94	348.25	148.95	35.04	58.81	24.00
10	91.76	22.21	49.70	12.24	45.90	15.54	130.16	38.99	109.78	52.33
11	46.87	13.07	18.35	7.90	422.74	684.73	69.16	68.08	115.07	37.51
12	56.61	30.16	26.83	7.18	23.64	21.01	77.90	20.37	95.89	47.55
13	557.15	219.99	244.72	125.98	439.23	125.01	326.17	239.48	151.17	109.91
14	580.95	167.07	313.28	123.02	164.89	31.09	602.62	198.57	248.32	100.61
15	214.32	111.31	105.87	28.28	365.01	119.93	142.41	71.53	534.27	292.48
16	237.36	48.29	161.47	98.67	207.95	43.53	221.14	59.16	235.23	53.25
17	80.17	31.75	67.81	46.11	58.35	28.55	62.51	20.24	38.40	16.82
18	54.81	41.22	23.90	17.82	41.83	20.97	40.23	20.48	48.03	28.25
19	105.95	28.96	64.73	21.24	83.66	30.39	96.60	32.46	127.95	22.23
20	280.12	221.01	150.68	75.94	113.56	74.10	489.76	677.23	132.26	38.39
21	51.71	26.92	25.90	8.06	69.19	32.14	160.84	38.05	299.71	158.75
22	231.45	122.50	119.90	90.49	132.61	62.31	200.30	164.81	511.30	762.30
23	196.97	116.29	109.93	61.33	91.94	57.19	43.08	30.80	44.54	21.35
24	73.70	14.05	56.95	27.58	75.95	23.34	142.82	57.15	108.83	26.71
25	577.04	146.54	319.33	84.47	262.23	74.47	324.68	68.78	276.31	72.60
26	316.27	68.25	128.10	37.11	126.48	66.99	210.26	72.89	184.77	71.26
27	150.19	61.09	96.52	29.51	253.61	64.35	91.78	32.88	176.32	52.49

Table S7. Within-subject means μ and standard deviations σ of five repeated measurements for HF in each experimental condition.

LF / HF

Participant ID	Experiment 1						Experiment 2			
	Rest 1		Standing		Cognitive Task 1		Rest 2		Cognitive Task 2	
	μ	σ	μ	σ	μ	σ	μ	σ	μ	σ
1	1.641	0.737	3.366	1.045	2.120	1.126	1.585	0.356	1.862	0.718
2	7.199	5.323	8.509	5.286	3.893	0.426	5.658	1.477	4.261	1.456
3	0.882	0.389	1.633	0.559	1.240	0.507	0.753	0.133	1.195	0.357
4	6.676	3.553	13.637	2.579	8.477	2.532	6.943	2.347	5.777	2.495
5	2.324	0.828	4.125	1.843	4.589	2.757	1.951	0.585	2.209	0.726
6	1.925	0.749	4.150	0.300	1.483	0.413	1.579	0.289	1.499	0.234
7	5.250	2.961	6.651	2.678	1.765	0.564	5.552	2.231	3.355	2.134
8	7.586	6.520	11.318	5.381	3.916	0.122	6.918	2.887	4.287	1.540
9	5.959	1.706	9.526	9.544	3.308	1.479	3.350	1.390	7.605	1.082
10	5.390	0.625	10.207	1.973	8.792	2.465	5.415	1.538	5.367	2.217
11	8.680	2.412	25.250	3.961	5.767	2.371	13.807	8.845	7.001	1.780
12	1.394	0.532	1.859	0.640	1.880	1.086	1.048	0.573	0.795	0.376
13	3.529	1.852	3.557	1.322	1.255	0.805	3.483	0.940	3.394	0.684
14	0.614	0.111	5.374	4.635	1.530	0.347	0.323	0.072	0.869	0.170
15	2.485	1.007	5.791	0.458	2.126	0.836	3.611	2.606	3.108	1.346
16	3.020	0.822	3.611	1.255	1.600	0.357	3.858	1.127	1.862	0.515
17	3.929	2.076	4.380	1.707	1.684	0.370	4.351	1.869	3.809	0.923
18	4.139	2.491	8.317	3.212	7.147	1.647	9.148	2.936	9.212	2.776
19	11.001	1.411	15.804	4.039	5.204	2.993	13.805	4.859	3.454	0.755
20	3.204	1.399	4.527	1.573	3.160	1.590	2.185	0.641	3.229	0.856
21	4.155	1.600	7.577	2.451	2.919	1.347	2.949	1.191	2.377	0.996
22	2.700	1.248	6.648	2.660	2.723	0.787	4.564	2.142	3.111	0.786
23	2.341	0.842	5.792	2.430	3.840	0.798	7.055	2.962	8.617	3.067
24	3.271	0.705	2.603	0.831	2.258	0.415	3.724	1.267	2.605	0.487
25	2.300	0.799	3.205	1.303	1.924	0.412	2.134	0.217	2.278	0.637
26	1.222	0.463	3.787	1.414	2.611	0.815	1.377	0.650	2.336	1.063
27	3.339	1.490	8.361	1.526	1.911	0.580	3.815	1.481	2.360	0.521

Table S8. Within-subject means μ and standard deviations σ of five repeated measurements for LF/HF in each experimental condition.

LFnorm

Participant ID	Experiment 1						Experiment 2			
	Rest 1		Standing		Cognitive Task 1		Rest 2		Cognitive Task 2	
	μ	σ	μ	σ	μ	σ	μ	σ	μ	σ
1	59.772	8.646	75.892	5.137	63.001	14.425	60.531	5.708	63.015	8.211
2	79.535	16.733	82.952	14.514	79.408	1.794	84.231	3.451	79.447	5.667
3	44.520	11.660	59.364	12.545	53.058	10.231	42.653	4.169	53.417	6.366
4	84.096	6.791	92.931	1.351	88.618	3.165	86.333	3.809	82.996	6.216
5	68.100	7.429	78.406	6.035	75.969	13.213	64.727	7.067	67.073	8.057
6	63.515	9.324	80.513	1.185	58.615	6.730	60.757	4.169	59.644	3.688
7	80.391	8.305	85.306	4.685	62.325	7.558	82.742	6.164	70.787	14.553
8	79.632	11.813	85.304	15.703	79.646	0.507	85.358	5.615	78.210	10.103
9	84.721	3.769	81.843	11.955	73.340	10.751	74.791	7.047	88.179	1.590
10	84.182	1.730	90.766	1.788	89.166	2.521	83.392	4.311	82.275	6.148
11	88.909	3.099	96.101	0.598	81.067	12.243	90.714	5.238	86.870	2.867
12	55.141	14.076	62.986	9.320	59.768	15.541	47.559	13.066	41.629	12.908
13	74.116	9.852	76.223	6.403	51.386	12.168	76.580	5.471	76.613	4.072
14	37.750	4.151	72.367	17.355	59.559	6.700	24.161	4.303	46.049	5.011
15	69.137	7.755	85.205	1.018	66.115	7.262	71.208	14.213	72.135	11.249
16	73.905	6.035	76.555	6.615	60.775	5.564	78.345	4.724	64.056	5.631
17	76.673	7.682	79.011	7.832	61.994	5.493	78.714	7.914	78.378	4.433
18	76.425	9.279	87.404	5.604	87.217	2.533	89.365	2.778	89.503	2.637
19	91.531	1.167	93.499	2.313	80.888	6.486	92.694	1.698	76.779	4.617
20	72.328	12.207	80.415	5.500	72.630	9.169	67.069	7.755	75.503	4.247
21	77.994	8.932	87.191	4.083	71.459	9.096	72.673	6.861	67.859	8.793
22	70.401	7.902	84.917	6.084	71.979	5.598	78.258	10.668	74.847	4.394
23	68.442	6.569	82.906	7.262	78.762	3.568	85.753	5.100	88.563	3.479
24	75.741	5.134	70.644	7.001	68.837	3.727	77.142	6.442	71.752	3.839
25	67.975	7.210	73.999	7.599	64.993	5.738	67.939	2.211	68.395	5.721
26	53.341	7.987	77.431	5.784	70.676	7.355	55.553	8.703	66.832	10.321
27	73.934	9.127	89.048	1.658	64.055	8.091	77.484	5.973	69.515	4.729

Table S9. Within-subject means μ and standard deviations σ of five repeated measurements for LFnorm in each experimental condition.

HFnorm

Participant ID	Experiment 1						Experiment 2			
	Rest 1		Standing		Cognitive Task 1		Rest 2		Cognitive Task 2	
	μ	σ	μ	σ	μ	σ	μ	σ	μ	σ
1	40.228	8.646	24.108	5.137	36.999	14.425	39.469	5.708	36.985	8.211
2	20.465	16.733	17.048	14.514	20.592	1.794	15.769	3.451	20.553	5.667
3	55.480	11.660	40.636	12.545	46.942	10.231	57.347	4.169	46.583	6.366
4	15.904	6.791	7.069	1.351	11.382	3.165	13.667	3.809	17.004	6.216
5	31.900	7.429	21.594	6.035	24.031	13.213	35.273	7.067	32.927	8.057
6	36.485	9.324	19.487	1.185	41.385	6.730	39.243	4.169	40.356	3.688
7	19.609	8.305	14.694	4.685	37.675	7.558	17.258	6.164	29.213	14.553
8	20.368	11.813	14.696	15.703	20.354	0.507	14.642	5.615	21.790	10.103
9	15.279	3.769	18.157	11.955	26.660	10.751	25.209	7.047	11.821	1.590
10	15.818	1.730	9.234	1.788	10.834	2.521	16.608	4.311	17.725	6.148
11	11.091	3.099	3.899	0.598	18.933	12.243	9.286	5.238	13.130	2.867
12	44.859	14.076	37.014	9.320	40.232	15.541	52.441	13.066	58.371	12.908
13	25.884	9.852	23.777	6.403	48.614	12.168	23.420	5.471	23.387	4.072
14	62.250	4.151	27.633	17.355	40.441	6.700	75.839	4.303	53.951	5.011
15	30.863	7.755	14.795	1.018	33.885	7.262	28.792	14.213	27.865	11.249
16	26.095	6.035	23.445	6.615	39.225	5.564	21.655	4.724	35.944	5.631
17	23.327	7.682	20.989	7.832	38.006	5.493	21.286	7.914	21.622	4.433
18	23.575	9.279	12.596	5.604	12.783	2.533	10.635	2.778	10.497	2.637
19	8.469	1.167	6.501	2.313	19.112	6.486	7.306	1.698	23.221	4.617
20	27.672	12.207	19.585	5.500	27.370	9.169	32.931	7.755	24.497	4.247
21	22.006	8.932	12.809	4.083	28.541	9.096	27.327	6.861	32.141	8.793
22	29.599	7.902	15.083	6.084	28.021	5.598	21.742	10.668	25.153	4.394
23	31.558	6.569	17.094	7.262	21.238	3.568	14.247	5.100	11.437	3.479
24	24.259	5.134	29.356	7.001	31.163	3.727	22.858	6.442	28.248	3.839
25	32.025	7.210	26.001	7.599	35.007	5.738	32.061	2.211	31.605	5.721
26	46.659	7.987	22.569	5.784	29.324	7.355	44.447	8.703	33.168	10.321
27	26.066	9.127	10.952	1.658	35.945	8.091	22.516	5.973	30.485	4.729

Table S10. Within-subject means μ and standard deviations σ of five repeated measurements for HFnorm in each experimental condition.

ApEn

Participant ID	Experiment 1						Experiment 2			
	Rest 1		Standing		Cognitive Task 1		Rest 2		Cognitive Task 2	
	μ	σ	μ	σ	μ	σ	μ	σ	μ	σ
1	1.1178	0.1713	0.9707	0.0494	1.2085	0.0818	1.2343	0.0365	1.2359	0.0638
2	0.9802	0.1466	0.7369	0.2266	1.2237	0.0878	1.1750	0.0512	1.1809	0.0596
3	1.1345	0.0679	1.0243	0.1116	1.1749	0.0970	1.0509	0.0929	1.1011	0.1603
4	1.1263	0.0965	0.8105	0.0766	1.0561	0.0694	0.9488	0.0826	1.1396	0.1223
5	1.2310	0.0194	1.1515	0.0825	1.2135	0.0613	1.2082	0.0408	1.1715	0.0377
6	1.3277	0.0352	1.2279	0.0540	1.3926	0.0491	1.2701	0.0209	1.2945	0.0693
7	1.0073	0.0903	0.8700	0.1041	1.2407	0.0765	1.0150	0.1044	0.9650	0.3575
8	1.0349	0.3032	0.5335	0.0733	1.1973	0.0314	0.9333	0.1256	1.1388	0.1713
9	1.0053	0.0901	0.6416	0.1527	1.1446	0.2877	0.9591	0.1729	1.0157	0.0809
10	1.1228	0.0444	0.9808	0.0426	1.2613	0.0475	1.0087	0.0401	1.2336	0.0321
11	0.9955	0.1954	0.7854	0.0974	1.1734	0.0931	0.9705	0.1015	1.2407	0.0677
12	1.2087	0.0499	1.2176	0.0342	1.2086	0.0346	1.1940	0.0309	1.2771	0.0415
13	0.9628	0.1257	0.8526	0.1418	1.2617	0.1491	0.9320	0.1043	1.1670	0.0726
14	1.2782	0.0366	1.1142	0.1129	1.3180	0.0260	1.2045	0.0328	1.3055	0.0058
15	1.2425	0.0320	1.0774	0.1608	1.2601	0.0470	1.1957	0.1263	1.2281	0.0620
16	1.1110	0.1111	1.2560	0.0549	1.2733	0.0527	1.0760	0.0581	1.1983	0.1175
17	1.1171	0.1540	1.0486	0.1093	1.2058	0.0576	1.0171	0.0845	1.1521	0.0983
18	1.1567	0.1049	1.0695	0.0887	0.8035	0.2101	1.1146	0.1173	1.2166	0.1238
19	1.0744	0.0602	0.9069	0.1023	1.2497	0.0507	1.0341	0.0938	1.2407	0.0072
20	1.1527	0.1405	1.0477	0.0920	1.2926	0.0248	1.1927	0.1217	1.2188	0.0293
21	1.0328	0.1535	0.9976	0.0882	1.2059	0.0493	1.1417	0.1213	1.1853	0.0522
22	1.1403	0.0329	0.9241	0.0842	1.2542	0.0752	1.2202	0.0631	1.2114	0.0998
23	1.2641	0.0851	1.1759	0.0462	1.2469	0.0564	1.0774	0.0992	1.0344	0.0993
24	1.0536	0.0754	1.0425	0.0873	1.2169	0.0192	1.1284	0.0741	1.2000	0.0508
25	1.1887	0.0327	1.1350	0.0807	1.2834	0.0252	1.2133	0.0340	1.2821	0.0461
26	1.2561	0.0254	1.2603	0.0991	1.3148	0.0363	1.2690	0.0487	1.3275	0.0350
27	1.1978	0.0354	1.1255	0.0554	1.2359	0.0344	1.1945	0.0454	1.2407	0.0533

Table S11. Within-subject means μ and standard deviations σ of five repeated measurements for ApEn in each experimental condition.

SampEn

Participant ID	Experiment 1						Experiment 2			
	Rest 1		Standing		Cognitive Task 1		Rest 2		Cognitive Task 2	
	μ	σ	μ	σ	μ	σ	μ	σ	μ	σ
1	1.3629	0.3927	0.9585	0.0738	1.5422	0.2440	1.5904	0.1498	1.5989	0.2051
2	0.9822	0.1948	0.6864	0.2620	1.4676	0.1868	1.3517	0.1413	1.3385	0.1157
3	1.2424	0.1233	1.0737	0.1594	1.2689	0.1322	1.1058	0.1317	1.1739	0.2360
4	1.2037	0.1537	0.7602	0.1004	1.0881	0.1012	0.9325	0.0995	1.2580	0.2320
5	1.6138	0.0889	1.3635	0.2123	1.5469	0.2948	1.6467	0.1965	1.5587	0.1769
6	1.6132	0.1086	1.3289	0.1053	1.7949	0.1328	1.5251	0.0539	1.5485	0.1508
7	1.0882	0.1369	0.8593	0.1345	1.5167	0.1564	1.0982	0.1712	1.0751	0.4607
8	1.1440	0.4918	0.4623	0.0822	1.2844	0.0512	0.8766	0.1707	1.1809	0.2320
9	1.0211	0.1122	0.5859	0.1659	1.3624	0.4374	0.9477	0.2698	1.0609	0.1317
10	1.2070	0.0769	1.0340	0.0527	1.4737	0.0889	1.0732	0.0510	1.3947	0.0513
11	1.0662	0.2802	0.7583	0.1493	1.3230	0.1688	0.9893	0.1492	1.4401	0.1137
12	1.4464	0.1297	1.4353	0.0780	1.4828	0.0698	1.5055	0.1144	1.6625	0.2157
13	0.9945	0.2218	0.7987	0.1981	1.4983	0.3067	0.9127	0.1820	1.2553	0.1421
14	1.8870	0.2108	1.2183	0.2461	1.8592	0.0685	1.4537	0.1006	1.8347	0.1093
15	1.5352	0.0897	1.1679	0.2814	1.7275	0.0933	1.4379	0.2115	1.4645	0.1295
16	1.2318	0.2168	1.4423	0.1183	1.6254	0.0930	1.1252	0.0829	1.3955	0.2614
17	1.2306	0.2454	1.0504	0.1894	1.3499	0.1341	1.0590	0.1231	1.2655	0.2342
18	1.2626	0.1893	1.0909	0.1384	0.7638	0.2629	1.1746	0.1921	1.3371	0.2098
19	1.1796	0.1063	0.9124	0.1529	1.6015	0.1670	1.0944	0.1318	1.5392	0.0273
20	1.3586	0.2921	1.1182	0.1478	1.5877	0.0832	1.4027	0.2781	1.4332	0.0940
21	1.1535	0.2437	1.0428	0.1355	1.4997	0.1630	1.3446	0.2761	1.3291	0.1002
22	1.2970	0.0454	0.9523	0.1176	1.5444	0.1952	1.4457	0.1489	1.3739	0.2006
23	1.5567	0.1721	1.2964	0.0703	1.4795	0.1497	1.1508	0.1537	1.0540	0.1431
24	1.1196	0.1241	1.0923	0.1249	1.4141	0.0712	1.2421	0.1367	1.3916	0.1005
25	1.4829	0.1366	1.2736	0.1687	1.7693	0.0494	1.4845	0.1278	1.6616	0.1634
26	1.6610	0.1505	1.4930	0.1897	1.6689	0.1605	1.7144	0.1789	1.7072	0.1855
27	1.5355	0.1205	1.2647	0.1170	1.6664	0.0989	1.4312	0.0758	1.4833	0.1076

Table S12. Within-subject means μ and standard deviations σ of five repeated measurements for SampEn in each experimental condition.

Fractal Dimension

Participant ID	Experiment 1						Experiment 2			
	Rest 1		Standing		Cognitive Task 1		Rest 2		Cognitive Task 2	
	μ	σ	μ	σ	μ	σ	μ	σ	μ	σ
1	1.7813	0.0886	1.6378	0.0333	1.8264	0.0589	1.8390	0.0329	1.8195	0.0523
2	1.6946	0.0394	1.6221	0.0703	1.7776	0.0397	1.7202	0.0518	1.7623	0.0425
3	1.8233	0.0566	1.7036	0.0525	1.8048	0.0372	1.8309	0.0326	1.7891	0.0711
4	1.6952	0.0478	1.5619	0.0174	1.6937	0.0332	1.6247	0.0401	1.7270	0.0660
5	1.8105	0.0419	1.7282	0.0594	1.7940	0.0898	1.8363	0.0236	1.8734	0.0496
6	1.8158	0.0295	1.7723	0.0140	1.9071	0.0260	1.8192	0.0484	1.8381	0.0357
7	1.7785	0.0453	1.6256	0.0866	1.9009	0.0535	1.7257	0.0453	1.8218	0.0636
8	1.6528	0.1431	1.4282	0.0745	1.7320	0.0338	1.6191	0.0904	1.7203	0.0683
9	1.6492	0.0402	1.6030	0.0616	1.8150	0.0569	1.6703	0.0442	1.6632	0.0271
10	1.6733	0.0173	1.6579	0.0214	1.7595	0.0174	1.6850	0.0116	1.7251	0.0372
11	1.6544	0.0305	1.5546	0.0531	1.7461	0.0549	1.6323	0.0533	1.7655	0.0397
12	1.8017	0.0536	1.7761	0.0328	1.8938	0.0396	1.8822	0.0686	1.9193	0.0302
13	1.7419	0.0688	1.6636	0.0634	1.8525	0.0422	1.6960	0.0647	1.7521	0.0513
14	1.9201	0.0433	1.7935	0.0439	1.9394	0.0350	1.9198	0.0196	1.9622	0.0272
15	1.8066	0.0577	1.6532	0.0765	1.8822	0.0324	1.7522	0.1008	1.7946	0.0792
16	1.7309	0.0787	1.7480	0.0183	1.9047	0.0352	1.6602	0.0429	1.8721	0.0377
17	1.7130	0.0532	1.6031	0.0551	1.7883	0.0587	1.6474	0.0240	1.7223	0.0627
18	1.7443	0.0506	1.6494	0.0285	1.6056	0.0772	1.7244	0.0434	1.6878	0.0548
19	1.6480	0.0484	1.6128	0.0343	1.8050	0.0672	1.6046	0.0380	1.8093	0.0292
20	1.7856	0.0666	1.6911	0.0444	1.8098	0.0316	1.8152	0.0479	1.7930	0.0485
21	1.6967	0.0873	1.6183	0.0338	1.8091	0.0658	1.7246	0.1080	1.7968	0.0305
22	1.7547	0.0562	1.6364	0.0842	1.8063	0.0483	1.7563	0.0584	1.8199	0.0529
23	1.8372	0.0359	1.7143	0.0559	1.8282	0.0564	1.7382	0.0119	1.7271	0.0201
24	1.6837	0.0500	1.6753	0.0630	1.7756	0.0398	1.6785	0.0646	1.7376	0.0407
25	1.8138	0.0422	1.7628	0.0278	1.8829	0.0214	1.7602	0.0261	1.8316	0.0418
26	1.9029	0.0404	1.8175	0.0418	1.8728	0.0473	1.9612	0.0461	1.8718	0.0445
27	1.8050	0.0192	1.6966	0.0356	1.8856	0.0348	1.7809	0.0458	1.8139	0.0248

Table S13. Within-subject means μ and standard deviations σ of five repeated measurements for Fractal Dimension in each experimental condition.

SD1 / SD2

Participant ID	Experiment 1						Experiment 2			
	Rest 1		Standing		Cognitive Task 1		Rest 2		Cognitive Task 2	
	μ	σ	μ	σ	μ	σ	μ	σ	μ	σ
1	0.3145	0.1036	0.2020	0.0516	0.3839	0.1204	0.3852	0.0679	0.3903	0.0816
2	0.3219	0.2056	0.2962	0.2585	0.3249	0.0534	0.2796	0.0439	0.3384	0.0613
3	0.3249	0.1069	0.1895	0.0507	0.2713	0.0502	0.3269	0.0673	0.2870	0.1008
4	0.2317	0.0507	0.1594	0.0192	0.2110	0.0239	0.2353	0.0437	0.2906	0.0746
5	0.3626	0.0429	0.2801	0.0517	0.3883	0.1999	0.4041	0.0660	0.4458	0.0946
6	0.3615	0.0354	0.2616	0.0197	0.4564	0.0579	0.3649	0.0716	0.3945	0.0816
7	0.2971	0.0564	0.1640	0.0423	0.4575	0.1348	0.2606	0.0305	0.4407	0.2864
8	0.2179	0.1145	0.1692	0.1650	0.2402	0.0161	0.1770	0.0676	0.2466	0.0459
9	0.2024	0.0356	0.3427	0.2211	0.4803	0.2016	0.3012	0.0966	0.2217	0.0193
10	0.2318	0.0098	0.1952	0.0065	0.2880	0.0438	0.2294	0.0051	0.2598	0.0222
11	0.1891	0.0486	0.1492	0.0235	0.3733	0.2148	0.1846	0.0242	0.3084	0.0285
12	0.4176	0.2132	0.3038	0.0456	0.4268	0.0885	0.4642	0.1039	0.6323	0.1671
13	0.2854	0.0471	0.2839	0.0702	0.4040	0.0529	0.2456	0.0569	0.2924	0.0522
14	0.5914	0.1241	0.2754	0.0596	0.6009	0.0921	0.4744	0.0409	0.6506	0.0728
15	0.3427	0.0571	0.2229	0.0417	0.4685	0.0585	0.3150	0.0843	0.4574	0.1423
16	0.2621	0.0553	0.3525	0.1002	0.4967	0.0570	0.2077	0.0155	0.4271	0.0971
17	0.2369	0.0652	0.2428	0.0677	0.2992	0.0731	0.2162	0.0401	0.2973	0.0722
18	0.2282	0.0539	0.2066	0.0394	0.1420	0.0445	0.2084	0.0286	0.2452	0.0378
19	0.2129	0.0230	0.1748	0.0117	0.3616	0.0780	0.1908	0.0219	0.3313	0.0230
20	0.3583	0.0750	0.2568	0.0397	0.3243	0.0296	0.4418	0.2571	0.2959	0.0374
21	0.2159	0.0592	0.1824	0.0199	0.3498	0.0731	0.2648	0.0972	0.3923	0.1544
22	0.2682	0.0349	0.2394	0.1355	0.3181	0.0633	0.3052	0.0547	0.4771	0.2748
23	0.3879	0.0417	0.2654	0.0568	0.3745	0.0727	0.2468	0.0369	0.2511	0.0301
24	0.2115	0.0348	0.1897	0.0306	0.2926	0.0749	0.2487	0.0585	0.2813	0.0486
25	0.3532	0.0445	0.2858	0.0229	0.4908	0.0704	0.2970	0.0394	0.3602	0.0754
26	0.4875	0.0757	0.3197	0.0488	0.4713	0.1615	0.5745	0.1276	0.4843	0.0946
27	0.3433	0.0355	0.2326	0.0319	0.4515	0.0922	0.3072	0.0547	0.3410	0.0357

Table S14. Within-subject means μ and standard deviations σ of five repeated measurements for SD1/SD2 in each experimental condition.

CD										
Participant ID	**Experiment 1**						**Experiment 2**			
	**Rest 1**		**Standing**		**Cognitive Task 1**		**Rest 2**		**Cognitive Task 2**	
	μ	σ	μ	σ	μ	σ	μ	σ	μ	σ
1	1.9284	0.2888	1.6780	0.0637	2.0923	0.1971	2.1069	0.0948	2.1391	0.1119
2	1.6674	0.1634	1.4210	0.2699	2.0335	0.1118	1.9580	0.1040	1.9613	0.0623
3	1.9871	0.1471	1.7127	0.1889	1.9615	0.1349	1.8838	0.1407	1.9164	0.2409
4	1.8484	0.1418	1.5081	0.1298	1.8255	0.0729	1.6945	0.1026	1.9185	0.1752
5	2.1119	0.0599	1.9488	0.0984	2.0761	0.2207	2.1142	0.0928	2.1713	0.1205
6	2.1685	0.0524	1.9563	0.0736	2.2906	0.0745	2.0973	0.0691	2.1108	0.0830
7	1.9281	0.0748	1.6098	0.1490	2.1599	0.1003	1.8852	0.0615	1.7203	0.4350
8	1.7358	0.3798	1.1200	0.0746	1.9327	0.0370	1.6356	0.2015	1.9198	0.1719
9	1.7387	0.1173	1.3539	0.2191	1.9839	0.3802	1.7028	0.2020	1.7999	0.0781
10	1.8944	0.0624	1.7871	0.0280	2.0362	0.0722	1.9088	0.0347	1.9748	0.0145
11	1.7178	0.2444	1.5137	0.1631	1.9450	0.0982	1.6203	0.1296	2.0417	0.0694
12	2.0255	0.0965	2.0131	0.0930	2.1293	0.0871	2.1630	0.1232	2.2549	0.0874
13	1.9104	0.1561	1.6856	0.1553	2.2055	0.1109	1.7898	0.1705	2.0039	0.0769
14	2.3328	0.0493	1.9248	0.1655	2.3633	0.0148	2.2361	0.0287	2.3870	0.0392
15	2.0819	0.1076	1.7898	0.1803	2.2576	0.0478	2.0093	0.2012	2.0955	0.0906
16	1.9214	0.1326	2.0033	0.0730	2.2483	0.0552	1.7883	0.0627	2.1325	0.1543
17	1.8123	0.2255	1.7406	0.1822	2.0031	0.1265	1.7092	0.1246	1.9349	0.1501
18	1.8662	0.1710	1.7488	0.1137	1.4550	0.2188	1.8011	0.1227	1.9346	0.1311
19	1.8116	0.0772	1.6633	0.0832	2.1471	0.1398	1.7720	0.0937	2.1153	0.0485
20	1.9523	0.1839	1.7586	0.0898	2.0816	0.0636	1.9481	0.1798	2.0132	0.0667
21	1.7326	0.2297	1.6645	0.1108	2.0705	0.1240	1.9279	0.2189	1.9789	0.0993
22	1.9642	0.0935	1.7050	0.1338	2.0577	0.1175	2.0395	0.0976	2.0575	0.1422
23	2.1511	0.0822	1.9442	0.0862	2.1504	0.0985	1.8886	0.1062	1.8318	0.1003
24	1.7789	0.1000	1.7307	0.1414	1.9934	0.0789	1.8493	0.1180	1.9320	0.0843
25	2.0795	0.0450	1.9721	0.0948	2.2952	0.0405	2.0171	0.0740	2.1437	0.1137
26	2.2558	0.0814	2.0862	0.1288	2.2328	0.1018	2.3241	0.0781	2.3032	0.1003
27	2.0110	0.0666	1.8604	0.0929	2.1949	0.0630	2.0154	0.1056	2.1172	0.0585

Table S15. Within-subject means μ and standard deviations σ of five repeated measurements for CD in each experimental condition.

ICD

Participant ID	Experiment 1						Experiment 2			
	**Rest 1**		**Standing**		**Cognitive Task 1**		**Rest 2**		**Cognitive Task 2**	
	μ	σ	μ	σ	μ	σ	μ	σ	μ	σ
1	1.3199	0.2463	1.1061	0.0603	1.4724	0.1955	1.5033	0.0805	1.5363	0.1240
2	1.0898	0.1514	0.8734	0.2448	1.4315	0.0988	1.3496	0.0855	1.3437	0.0595
3	1.3989	0.1060	1.1467	0.1584	1.3750	0.1325	1.2996	0.1409	1.3473	0.2355
4	1.2585	0.1289	0.9613	0.1111	1.2340	0.0652	1.1158	0.0867	1.3183	0.1608
5	1.4726	0.0467	1.3533	0.1010	1.4610	0.2208	1.4877	0.0720	1.5716	0.1037
6	1.5460	0.0356	1.3466	0.0632	1.6725	0.0775	1.4839	0.0606	1.5020	0.0838
7	1.3312	0.0502	1.0393	0.1185	1.5487	0.0882	1.3046	0.0700	1.1467	0.3947
8	1.1805	0.3549	0.6459	0.0610	1.3376	0.0416	1.0822	0.1840	1.3228	0.1462
9	1.1703	0.1035	0.8305	0.1848	1.4059	0.3581	1.1250	0.1844	1.2236	0.0788
10	1.2976	0.0620	1.1985	0.0103	1.4399	0.0797	1.3145	0.0277	1.3868	0.0133
11	1.1417	0.2223	0.9626	0.1302	1.3238	0.1075	1.0478	0.1147	1.4408	0.0650
12	1.4115	0.0814	1.4086	0.0844	1.5098	0.0630	1.5541	0.1103	1.6366	0.0889
13	1.3410	0.1456	1.1297	0.1339	1.5984	0.1089	1.2247	0.1547	1.4000	0.0666
14	1.6933	0.0487	1.3402	0.1373	1.7284	0.0184	1.6442	0.0393	1.7608	0.0303
15	1.4478	0.1093	1.2166	0.1646	1.6174	0.0391	1.3904	0.1787	1.4782	0.0906
16	1.3411	0.1282	1.3852	0.0790	1.6383	0.0524	1.2114	0.0585	1.5115	0.1833
17	1.2303	0.2211	1.1623	0.1613	1.4211	0.1066	1.1308	0.1134	1.3413	0.1333
18	1.2601	0.1607	1.1591	0.0922	0.9178	0.2034	1.1887	0.1200	1.3466	0.1171
19	1.2193	0.0666	1.0727	0.0683	1.5207	0.1293	1.1890	0.0836	1.4996	0.0341
20	1.3572	0.1734	1.1815	0.0768	1.4817	0.0639	1.3585	0.1698	1.3883	0.0697
21	1.1536	0.2031	1.0948	0.0990	1.4619	0.1166	1.3338	0.2000	1.3787	0.0877
22	1.3713	0.0787	1.1428	0.1325	1.4680	0.1214	1.4367	0.0920	1.4660	0.1294
23	1.5400	0.0833	1.3431	0.0893	1.5517	0.1034	1.2989	0.0986	1.2303	0.0743
24	1.1911	0.0821	1.1403	0.1149	1.3856	0.0751	1.2639	0.1072	1.3466	0.0578
25	1.4682	0.0393	1.3650	0.0738	1.6614	0.0625	1.4115	0.0503	1.5301	0.1163
26	1.6633	0.0645	1.4880	0.1299	1.6174	0.1231	1.6899	0.0879	1.6868	0.0957
27	1.3964	0.0593	1.2627	0.0831	1.5784	0.0538	1.4035	0.1055	1.5286	0.0522

Table S16. Within-subject means μ and standard deviations σ of five repeated measurements for ICD in each experimental condition.

Index	(1) Rest 1 → Standing					(2) Rest 1 → Cognitive Task 1					(3) Rest 2 → Cognitive Task 2				
	p-value	Z	r	d	1-β	p-value	Z	r	d	1-β	p-value	Z	r	d	1-β
SDNN	0.0593	1.898	0.3653	0.3612	0.1942	2.26E-04	3.700	0.7120	0.9037	0.9552	0.0697	1.826	0.3514	0.3171	0.1420
SDSD	1.70E-04	3.772	0.7259	0.9048	0.9557	0.331	0.985	0.1896	0.0545	0.0125	0.264	1.129	0.2173	0.2823	0.1083
RMSSD	1.70E-04	3.772	0.7259	0.9048	0.9557	0.331	0.985	0.1896	0.0545	0.0126	0.264	1.129	0.2173	0.2823	0.1083
pNN50	6.65E-06	4.517	0.8692	1.0161	0.9870	0.657	0.456	0.0878	0.0778	0.0153	0.355	0.937	0.1803	0.2328	0.0708
LF	0.838	0.216	0.0416	0.0831	0.0161	5.52E-03	2.787	0.5363	0.5160	0.4482	0.801	0.264	0.0509	0.1710	0.0392
HF	3.60E-04	3.580	0.6889	0.6143	0.6308	0.0204	2.330	0.4485	0.3405	0.1684	0.971	0.048	0.0092	0.1337	0.0268
LF/HF	1.04E-05	4.421	0.8507	0.9422	0.9699	0.199	1.297	0.2497	0.2857	0.1113	0.0903	1.706	0.3283	0.3231	0.1485
LFnorm	2.48E-05	4.228	0.8138	1.1505	0.9979	0.524	0.649	0.1248	0.1288	0.0255	0.394	0.865	0.1665	0.0721	0.0145
HFnorm	2.48E-05	4.228	0.8138	1.1505	0.9979	0.524	0.649	0.1248	0.1288	0.0255	0.394	0.865	0.1665	0.0721	0.0145
ApEn	5.16E-05	4.060	0.7814	1.0361	0.9898	4.73E-04	3.508	0.6750	0.7147	0.7904	1.27E-04	3.844	0.7398	0.9191	0.9616
SampEn	2.00E-05	4.276	0.8230	1.4172	1.0000	6.75E-04	3.412	0.6566	0.7952	0.8824	8.04E-04	3.363	0.6473	0.8210	0.9046
Fractal Dimension	8.33E-06	4.469	0.8600	1.6845	1.0000	3.95E-04	3.556	0.6843	0.9157	0.9603	9.55E-04	3.315	0.6381	0.7139	0.7892
SD1/SD2	4.32E-04	3.532	0.6797	0.7983	0.8852	9.55E-04	3.315	0.6381	0.7903	0.8777	2.37E-03	3.051	0.5872	0.7027	0.7737
CD	1.04E-05	4.421	0.8507	1.4586	1.0000	4.73E-04	3.508	0.6750	0.8682	0.9371	1.70E-04	3.772	0.7259	0.8512	0.9265
ICD	7.44E-06	4.493	0.8646	1.5268	1.0000	3.95E-04	3.556	0.6843	0.9204	0.9622	1.70E-04	3.772	0.7259	0.8799	0.9436

Index	(4) Rest 1 → Rest 2					(5) Cognitive Task 1 → Cognitive Task 2				
	p-value	Z	r	d	1-β	p-value	Z	r	d	1-β
SDNN	0.355	0.937	0.1803	0.1237	0.0242	6.39E-03	2.739	0.5271	0.5705	0.5506
SDSD	0.728	0.360	0.0694	0.0032	0.0100	1.53E-01	1.441	0.2774	0.3222	0.1476
RMSSD	0.728	0.360	0.0694	0.0032	0.0100	0.153	1.441	0.2774	0.3222	0.1476
pNN50	0.952	0.072	0.0139	0.1028	0.0195	0.153	1.441	0.2774	0.1453	0.0302
LF	0.307	1.033	0.1988	0.0196	0.0103	2.06E-04	3.724	0.7167	0.2754	0.1023
HF	0.857	0.192	0.0370	0.1302	0.0259	0.0355	2.114	0.4069	0.2904	0.1156
LF/HF	0.407	0.841	0.1618	0.2907	0.1158	0.307	1.033	0.1988	0.1759	0.0412
LFnorm	0.368	0.913	0.1757	0.1429	0.0295	0.343	0.961	0.1849	0.1861	0.0456
HFnorm	0.368	0.913	0.1757	0.1429	0.0295	0.343	0.961	0.1849	0.1861	0.0456
ApEn	0.153	1.441	0.2774	0.2325	0.0706	0.0263	2.234	0.4300	0.2262	0.0667
SampEn	0.121	1.562	0.3005	0.2806	0.1068	0.0105	2.571	0.4947	0.4329	0.3005
Fractal Dimension	0.274	1.105	0.2127	0.2560	0.0869	0.0335	2.138	0.4115	0.4622	0.3502
SD1/SD2	0.838	0.216	0.0416	0.0646	0.0136	0.464	0.745	0.1433	0.1212	0.0236
CD	0.254	1.153	0.2219	0.1623	0.0359	0.0735	1.802	0.3468	0.2922	0.1172
ICD	0.254	1.153	0.2219	0.1484	0.0312	0.0815	1.754	0.3375	0.2793	0.1056

Table S17. Summary of post hoc power analyses for all HRV indices. Reported values include p-values, standardised test statistics (Z) derived from the Wilcoxon signed-rank test, rank-based effect sizes (r), difference-based effect sizes (d), and achieved power ($1-\beta$) for each comparison. The estimations of d and $1-\beta$ are approximate and based on the assumption of normality of paired differences; the sample size $N = 27$. For the post hoc power analyses, a significance level of $\alpha = 0.01$ (i.e., before adjustment by the Holm–Bonferroni method) was assumed.
